# Sleep restoration by optogenetic targeting of GABAergic neurons reprograms microglia and ameliorates pathological phenotypes in an Alzheimer’s disease model

**DOI:** 10.1186/s13024-023-00682-9

**Published:** 2023-12-01

**Authors:** Qiuchen Zhao, Megi Maci, Morgan R. Miller, Heng Zhou, Fang Zhang, Moustafa Algamal, Yee Fun Lee, Steven S. Hou, Stephen J. Perle, Hoang Le, Alyssa N. Russ, Eng H. Lo, Dmitry Gerashchenko, Stephen N. Gomperts, Brian J. Bacskai, Ksenia V. Kastanenka

**Affiliations:** 1 Department of Neurology, MassGeneral Institute of Neurodegenerative Diseases, Massachusetts General Hospital, Harvard Medical School, Charlestown, MA 02129 USA; 2 Departments of Radiology, Massachusetts General Hospital, Harvard Medical School, Charlestown, MA 02129 USA; 3 Department of Psychiatry, Harvard Medical School and Veterans Affairs Boston Healthcare System, West Roxbury, MA 02132 USA

**Keywords:** Optogenetics, Microglia, EEG, Sleep, Slow oscillations, Alzheimer’s disease, Multiphoton microscopy, Memory consolidation, Sleep deprivation

## Abstract

**Background:**

Alzheimer’s disease (AD) patients exhibit memory disruptions and profound sleep disturbances, including disruption of deep non-rapid eye movement (NREM) sleep. Slow-wave activity (SWA) is a major restorative feature of NREM sleep and is important for memory consolidation.

**Methods:**

We generated a mouse model where GABAergic interneurons could be targeted in the presence of APPswe/PS1dE9 (APP) amyloidosis, APP-GAD-Cre mice. An electroencephalography (EEG) / electromyography (EMG) telemetry system was used to monitor sleep disruptions in these animals. Optogenetic stimulation of GABAergic interneurons in the anterior cortex targeted with channelrhodopsin-2 (ChR2) allowed us to examine the role GABAergic interneurons play in sleep deficits. We also examined the effect of optogenetic stimulation on amyloid plaques, neuronal calcium as well as sleep-dependent memory consolidation. In addition, microglial morphological features and functions were assessed using confocal microscopy and flow cytometry. Finally, we performed sleep deprivation during optogenetic stimulation to investigate whether sleep restoration was necessary to slow AD progression.

**Results:**

APP-GAD-Cre mice exhibited impairments in sleep architecture including decreased time spent in NREM sleep, decreased delta power, and increased sleep fragmentation compared to nontransgenic (NTG) NTG-GAD-Cre mice. Optogenetic stimulation of cortical GABAergic interneurons increased SWA and rescued sleep impairments in APP-GAD-Cre animals. Furthermore, it slowed AD progression by reducing amyloid deposition, normalizing neuronal calcium homeostasis, and improving memory function. These changes were accompanied by increased numbers and a morphological transformation of microglia, elevated phagocytic marker expression, and enhanced amyloid β (Aβ) phagocytic activity of microglia. Sleep was necessary for amelioration of pathophysiological phenotypes in APP-GAD-Cre mice.

**Conclusions:**

In summary, our study shows that optogenetic targeting of GABAergic interneurons rescues sleep, which then ameliorates neuropathological as well as behavioral deficits by increasing clearance of Aβ by microglia in an AD mouse model.

**Supplementary Information:**

The online version contains supplementary material available at 10.1186/s13024-023-00682-9.

## Background

Alzheimer’s disease (AD), the most common cause of dementia, is associated with sleep disruptions as early as the preclinical stages of the disease [1, 2]. Sleep fragmentations and disruptions of non-rapid eye movement (NREM) sleep are prevalent during AD [3, 4]. NREM sleep is characterized by high-voltage synchronized slow-wave activity (SWA) throughout the cortex and is referred to as slow-wave sleep in its most synchronized form [5–7]. SWA is important for memory consolidation during sleep and is associated with proper cognitive function [8]. Disruptions in SWA occur early during the disease progression [3]. Amyloid plaque burden positively correlates with impaired NREM sleep as well as aberrant SWA during sleep [1, 3, 9–12]. APPswe/PS1dE9 (APP) mice [13], a mouse model of amyloidosis, exhibit profound NREM sleep disruptions and SWA impairments prior to the appearance of amyloid plaques, yet in the presence of oligomeric amyloid β (Aβ). SWA impairments occur prior to the exhibition of memory deficits [14–16]. Therefore, there is an urgent need to ameliorate sleep deficits, determine whether it can also slow AD progression, and ultimately rescue memory function.

A number of studies reported that GABAergic interneurons were important for sleep generation and its regulation, particularly for the generation of SWA during healthy NREM sleep [17–19]. Our previous work reported SWA deficits in APP mice. These mice exhibited deterioration of SWA power, low cortical protein levels of Gamma-aminobutyric acid (GABA) and GABA receptors, GABA_A_ as well as GABA_B_. SWA disruption was due to circuit hyperactivity as the application of GABA directly to cortices of APP mice rescued SWA [14]. GABA signaling was necessary and sufficient for the generation of slow waves, and impaired inhibitory elements of the cortico-thalamic circuit were indicated as the cause of SWA disruption in APP mice. Thus, improving sleep by targeting inhibitory interneurons holds promise for slowing disease progression.

Sleep is essential for a healthy immune system [20–22]. The immune-supportive function of sleep is thought to be primarily conveyed by NREM sleep [23, 24]. Microglia are innate immune cells of the central nervous system (CNS) that are derived from myeloid progenitor cells [25]. In addition to their classical immune cell function, microglia act as guardians of the brain by promoting phagocytic clearance and providing trophic support to ensure tissue repair as well as maintenance of cerebral homeostasis. Evidence suggests microglial-mediated Aβ clearance is compromised in AD [26]. Recent studies have documented the effects of sleep loss on Aβ clearance, microglial morphology, and phagocytosis [27–29]. Impairment of the sleep − wake cycle diminishes microglial-mediated clearance of Aβ [25]. However, it remains unclear whether sleep restoration could reprogram disease-associated microglial response and improve its Aβ clearance ability.

In this study, we investigated the sleep architecture of APP-GAD-Cre mice early in the disease progression (6 months of age) and compared to that of nontransgenic (NTG) NTG-GAD-Cre controls. APP-GAD-Cre mice exhibited sleep impairments including decreased time spent in NREM sleep, decreased delta power, and increased sleep fragmentation. Optogenetic targeting of GABAergic interneurons in the anterior cortex increased SWA and NREM sleep. Chronic optogenetic stimulation slowed amyloid deposition and ameliorated neuronal calcium overload as well as memory deficits in APP-GAD-Cre mice. Furthermore, we observed increased numbers and a morphological transformation of microglia toward the phagocytic state. The treatment reprogrammed the microglial clearance ability and expression of CD68 and CSF-1R. Our results indicate that activation of the GABAergic interneurons ameliorates sleep disruptions, slows AD-related pathology and improves sleep-dependent memory consolidation in an AD mouse model.

## Methods

### Animals

The transgenic mouse line APPswe/PS1dE9 (APP), which co-expresses the Swedish mutation of amyloid precursor protein and a deltaE9 mutation in presenilin 1 [13] (The Jackson Laboratory, stock No. 034829), was compared to non-transgenic littermate control (NTG) mice matched for age. Homozygous GAD-Cre mice [30] (Gad2tm2(cre)Zjh/J; the Jackson Laboratory, stock No. 010802) were crossed with heterozygous APP mice to target GABAergic neuronal population in presence of amyloidosis. This cross resulted in APP-GAD-Cre and NTG-GAD-Cre progeny which were then used in the present studies. All methods employed in this study were conducted in compliance with IACUC and National Institutes of Health Guidelines for the Use of Laboratory Animals. In this study, both male and female mice were included. Animals were separated by sex and housed in cages with a maximum of 4 mice. Mice were housed individually following surgical procedures. Mice inhabited microdialysis bowls throughout the duration of chronic optogenetic treatments. All animals were granted free access to food and water ad libitum. Animals were maintained on a 12-hour light/12-hour dark cycle with lights on from 07:00 to 19:00 h in a pathogen-free environment.

### Stereotactic adeno-associated virus (AAV) infusions and cannula implantation

Intracortical viral injections were developed as previously described [14]. Briefly, APP-GAD-Cre and NTG-GAD-Cre mice were anesthetized with 4% isoflurane and placed on a stereotaxic frame. Once secured to the apparatus, isoflurane concentration was decreased to 1.5% and administered transnasally for the duration of surgical procedures. Following scalp disinfection, skin was incised down the midline. Intracortical viral injections were administered to the left anterior cortex with the following coordinates: AP + 1, ML + 0.5, DV − 0.9 at 0.1 µl/min. A 1-ml Hamilton syringe was employed to inject either a virus containing 1.5 µl of AAV-5-EF1a-DIO-hChR2(H134R)-mCherry (1 × 10^13^ virus molecules/ml, University of North Carolina) or a control vector AAV5-EF1a-DIO-mCherry (1 × 10^13^ virus molecules/ml, University of North Carolina) in absence of ChR2. Yellow Cameleon 3.6 (YC3.6) is a ratiometric indicator that enables visualization of basal neuronal calcium levels, which are elevated in AD [31, 32]. A viral vector containing 3 µl of AAV2-CBA-YC3.6 (University of Pennsylvania) was targeted to the right posterior cortex with the following coordinates: AP − 3, ML − 1, DV − 0.7 at 0.15 µl/min (2 × 10^12^ molecules/ml). The virus was then allowed to diffuse for 10 min before the injection needle was removed. After injections were completed, the skin was sutured, and the mice were maintained on a heating pad anesthesia-free until alert and mobile. Mice were allowed to rest for at least three weeks before initiating any experimental procedures to allow for proper recovery and viral expression. Viral expression was verified in all mice. A light-guide cannula (Doric Lenses) was placed and fixed over the site of viral expression. The cannula was installed over the cortex carefully. C&B Metabond (Parkell; item no. 242–3200) was used to secure two small screws installed at the anterior and posterior border of the surgical site to secure the cannula in place.

### Optogenetic stimulation protocol

Neurons in the anterior cortex were optogenetically manipulated following virus injections and cannula placements. 400 ms pulses of 473 nm light (5 to 7 mW) were delivered at 0.6 Hz, 24 h/day via a fiberoptic cable attached to each cannula for 14 or 28 days to restore the power of slow oscillations in each APP-GAD-Cre mouse. WT-GAD-Cre mice received identical treatment. A separate cohort of APP-GAD-Cre mice received 40 Hz stimulation by delivering 473 nm light (1 mW) to the anterior cortex continuously for 14 days. Mice individually inhabited microdialysis bowls (Harvard Apparatus, Holliston, MA) for the entirety of optogenetic treatment with food and water access ad libitum.

### Sleep recording

APP-GAD-Cre and NTG-GAD-Cre mice were implanted with wireless HD-X02 transmitters (bandwidth of 0.1 to 200 Hz for electroencephalography (EEG) and electromyography (EMG), DSI Harvard Bioscience, Minneapolis, MN) under isoflurane anesthesia (3% induction, 1.5% maintenance) for wireless EEG and EMG telemetry recordings. Transmitters were placed subcutaneously by an experienced researcher using aseptic techniques. The skull was exposed and cleaned. Two stainless steel screws (M06-15-M-SS-P; US Micro Screw, Seattle, WA) that served as cortical electrodes were passed through the skull to connect with the dura mater. One screw was installed 1 mm anterior to bregma, 1 mm lateral from the sagittal suture and the second screw was installed 3 mm posterior to bregma, 3 mm contralateral from the sagittal suture. EMG leads were fastened to muscles surrounding the neck. Before recording any data, the mice were given 10 days to recover from the procedure. EEG/EMG data, activity, and temperature data were collected using Ponemah Software v6.50 (DSI Harvard Bioscience, Minneapolis, MN) while animals were maintained in microdialysis bowls situated over the DSI RPC-1 receivers.

### Sleep analyses

Sleep scoring was performed using NeuroScore Software v3.3 (DSI Harvard Bioscience, Minneapolis, MN). In brief, raw telemetry signals including EEG, EMG, activity, and temperature were imported, bandpass filtered (EEG: 0.5–100 Hz; EMG: 10–100 Hz) and analyzed in 10-s epochs as either wake, rapid eye movement sleep (REM), or NREM sleep using the NeuroScore Mouse Sleep scoring module. The delta band included frequencies 0.5–4 Hz and the theta band included frequencies 6–9 Hz. Wake state was characterized by the combination of variable high-frequency EEG and heightened EMG activity. NREM sleep was characterized by the combination of low-frequency, high-amplitude EEG and diminished EMG activity. REM sleep was defined as a predominance of theta frequencies in the EEG (> 3 theta/delta ratio) and diminished EMG activity. A bout was characterized as at least two consecutive 10-s epochs of a sleep state. For spectral analyses, EEG signals were analyzed with a fast Fourier transform algorithm using a Hanning Window in NeuroScore without overlap on all epochs without artifact. Assessments of relative power enabled direct comparisons of EEG power spectra. Relative band power was calculated by quantifying both the band power and total power ratio. To compare power ratios, the ratio between band powers was computed.

### Multiphoton imaging and data acquisition

APP-GAD-Cre and NTG-GAD-Cre mice were anesthetized by inhalation of 1.5% isoflurane. A round craniotomy was made with a dental drill and an 8 mm glass coverslip was placed over the viral expression site. A mixture of dental cement and Krazy glue was used to secure the coverslip to the surrounding skull. Texas Red dextran was administered intravenously to the retro-orbital sinus for visualization of circulatory vessels. One day before imaging, mice were injected intraperitoneally with Methoxy-XO4 (10 mg/ kg), which binds to amyloid plaques, allowing for visualization through cranial windows with multiphoton microscopy. Mice were imaged using a FluoView FV1000MPE two-photon laser-scanning system (Olympus) mounted on a BX61WI microscope (Olympus), equipped with a long working distance 25x (numerical aperture = 1.05) dipping water immersion objective (Olympus). A mode-locked titanium/sapphire laser (MaiTai; Spectra-Physics, Fremont, CA) allowed for excitation at 800 or 860 nm to invoke two-photon fluorescence. For light collection, three photomultiplier tubes (PMTs, Hamamatsu, Ichinocho, Japan) individually detected wavelengths between 380 − 480 nm, 500 − 540 nm, and 560 − 650 nm. PMT settings were kept constant for each experiment, but laser power was adjusted as needed. Imaging of amyloid plaque pathology was acquired with photon excitation at 800 nm and a digital magnification of 1X. Neurites expressing YC3.6 were excited at 860 nm and imaged at a digital magnification of 2X. To prevent photobleaching, the laser power was maintained under 30mW. Z-stack images were sampled at intervals of 1–5 μm. A heating pad under the mice maintained appropriate, physiological body temperature throughout the duration of image acquisition.

### Image processing and data analysis

Image stacks acquired with multiphoton microscopy were analyzed using ImageJ. To determine amyloid plaque number and amyloid plaque burden, maximum intensity projections of each Z-stack were generated. Amyloid plaques were quantified and analyzed in APP-GAD-Cre mice. To calculate amyloid burden, projected images were thresholded and segmented such that the positive signal could be measured as a percentage of cortical area. Amyloid plaque quantification was represented by both number and burden with respect to cortical volume or area, respectively. ImageJ software was used to analyze all images of neuronal processes with expression of YC3.6 in both APP- and NTG-GAD-Cre mice. YC3.6 is a Fluorescence Resonance Energy Transfer (FRET) probe. The ratio of YFP to CFP provides an assessment of the calcium concentration within individual cells. A larger YFP:CFP ratio corresponds with higher concentrations of calcium [31]. The YFP images were used to manually classify neurites and draw each as regions of interest (ROI) with the “freehand” tool in ImageJ. YFP/CFP ratios were calculated and converted to [Ca^2+^] with standard equations using the *in-situ* Kd and Hill coefficient for YC3.6 determined previously [31]. Pseudocolored images were created in Matlab based on the YFP/CFP ratio, which was converted to calcium concentration using the empirical Rmin and Rmax and assigned to the jet colormap. The ratio values were used to supply the Hue and Saturation (color) and the reference image was used to supply the Value (intensity). Data are represented as histograms and percentages of neurites expressing calcium elevations or overload.

### Open field, Y-maze and contextual fear conditioning tests

Every APP-GAD-Cre and NTG-GAD-Cre mouse was habituated to the behavioral apparatus 1 day prior to the behavior tests. Mice were freely acclimated to the experimental room for a minimum of 1 h before testing. On the testing day, mice were individually situated in an arena with dimensions of 27 cm x 27 cm x 27 cm and granted to roam freely for 10 min during the open field test. Locomotor activity was recorded by EthoVision XT software (Noldus, Wageningen, the Netherlands), which tracked and analyzed all locomotor activity. Y-maze test was performed using a Y-shaped maze with three polycarbonate opaque arms orientated at 120 angles from each other. The mouse was placed in the center of the maze and allowed to explore the arms freely for 10 min. An entry was defined by all four limbs of the mouse being present within an arm. An alternation was defined as consecutive entries into distinct three arms. Quantity of arm entries and triads were documented for calculating the percentage of alternation.

Mice were individually situated in a fear conditioning chamber (30 × 24 × 21 cm; MED-Associates, St. Albans, VT) and recorded for 5 min with a camera. Mice were given 3 foot-shocks (1 s, 1 mA, 1 min interval) following 2 min of baseline recording. The fear conditioning chamber was enclosed by aluminum walls and had a clear, polycarbonate door. The foot-shocks were administered by a removable grid floor that contained 36 stainless steel grid rods (3.18 mm in diameter, 8.13 mm apart). Animals were placed in home cages and allowed to sleep after fear acquisition. On the day following fear acquisition, all mice were placed back in the conditioning chamber for fear recall (recording for 3 min) without foot-shock delivery. This allowed assessment of sleep-dependent memory (foot shock) consolidation. The threshold of freezing levels (i.e., the value of the motion index below which no movement is detectable) was determined. Fear memory was measured as the percentage of time that mice spent in the freezing state using Video Freeze software (Med associates Inc, Fairfax, VT).

The equipment was cleaned with 70% ethanol between animals to remove any scent left by the previous subject mouse.

### Flow cytometry and Aβ phagocytosis assay

APP-GAD-Cre mice received an i.p. injection of Methoxy-X04 to label fibrillar amyloid beta plaques. 3 h later, mice were anesthetized and perfused with 50 mL of ice-cold 1× PBS. Single-cell suspensions were prepared from whole brain tissue. Cell suspensions were centrifuged at 2,000 rpm for 5 min, and cell pellets were collected. Thereafter, 5 ml of 30% Percoll solution was used to resuspend the cell pellet. The gradient was centrifuged at 2,000 rpm for 30 min at room temperature. Cell pellets were collected for antibody staining. Then these cells were stained with CD11b-APC/Cy7, CD45-APC, CD36-PerCP/Cy5.5, CD68-BV711 and Colony-stimulating factor 1 receptor (CSF-1R)-PE/Cy7. All antibodies were purchased from Biolegend (San Diego, CA). Antibody incubations were performed according to manufacturer’s instructions. Samples were analyzed on a FACS FORTESSA flow cytometer (BD Bioscience, Franklin lakes, NJ). Data were analyzed with Flow Jo software version 7.6.1 (Flow J, LLC, Ashland, OR). WT mice injected with Methoxy-X04 were used as controls to determine the Methoxy threshold for non-phagocytosing cells.

### Immunohistochemistry

Fixation of the brain tissue was achieved by incubation of the freshly dissected mouse brains in 15% glycerol, 4% paraformaldehyde solution for 72-hours. Coronal Sect. (40 μm) were obtained using a Vibratome (Leica VT1000 S, Deer Park, IL) and stored in cryoprotectant at -20 °C. For confocal imaging of microglia, 40 μm coronal brain sections with Methoxy-XO4-labeled amyloid plaques underwent antigen retrieval with citrate buffer. Tissue sections were permeabilized with 0.5% Triton X-100 in TBS, blocked with normal goat serum, and incubated with Iba-1 (Rabbit monoclonal anti-Iba1, 1:200; 019-19741; Wako, Osaka, Japan) at 4 °C overnight. On the following day, sections were incubated with a corresponding secondary antibody (1:500) for 1 h at room temperature. Lastly, all slides were mounted, using the Vectashield antifade mounting media (Vector Laboratories) without DAPI. An inverted Olympus confocal microscope (Olympus FV3000RS Confocal Laster Scanning Microscope, Japan) with a 40X objective was used to acquire Z stack images for the purpose of morphological analysis. For amyloid plaque burden analysis, all sections similarly underwent antigen retrieval with citrate buffer. Sections were permeabilized with 0.5% Triton X-100 in TBS, blocked with normal goat serum, and incubated with 6E10 (Mouse monoclonal anti-6E10, 1:500; SIG-39,347; Covance) as well as 82E1 (Mouse anti-82E1, 1:500, IBL 10,323; Immuno-Biological Laboratories; Minneapolis, MN). Sections were then imaged using an inverted Zeiss microscope (Zeiss Axio Imager Z2, Germany) with a 10X objective. A positive signal threshold was determined such that amyloid burden could be calculated as occupying a fraction of either hippocampal or cortical area. Representative images were acquired with a 10X objective.

### Microglial morphology analysis

3D reconstruction was conducted with Imaris software (Bitplane, Switzerland) to analyze the microglial number, cell body volume, process length and clustering pattern around amyloid plaques in 40 μm brain sections. All images were preprocessed using the same threshold setting prior to analysis. The surfaces module was utilized to identify, and 3D render plaques (blue) based on the Methoxy-XO4 signal. Iba1-positive microglia (green) were then counted using the spots module, placing a sphere at the soma of each cell. Finally, the Spots Close To Surface XTension was conducted to distinguish plaque associated microglia. The subset of spots closer to plaque than the defined threshold (25 μm) was presented in the sphere (purple). Spots out of this range were excluded. The algorithm calculated the distance of each microglia to the nearest amyloid plaque in 3D space, allowing quantification of microglial aggregation around plaques.

### Sleep deprivation

Sleep deprivation consisted of gentle-handling as a method of sleep-deprivation in mice for 6 h, starting at 7 am until 12 pm daily for 28 days. Gentle handling is a widely accepted technique that minimally disturbs the ongoing activity of the animals while keeping them awake for prolonged periods of time. It consists of gentle cage-shaking and touching the animals with the hand or a soft brush. This protocol has been shown to be less stressful than other methods of sleep deprivation such as forced locomotion or electric shock [33]. Continuous optogenetic stimulation of GABAergic interneurons at 0.6 Hz as described above was performed during sleep-deprivation.

### Statistics

Statistical analyses were performed in GraphPad 8.0. Data were expressed as mean ± SEM. Datasets were tested for normality (Shapiro-Wilk normality test, D’Agostino & Pearson omnibus normality test or Kolmogorov-Smirnov test), after which appropriate statistical tests were used (t-test or ANOVA for normally distributed data, Mann-Whitney or Kruskal-Wallis test followed by Dunn’s multiple comparison test for nonparametric data). For datasets comparing 2 conditions, p < 0.05 was considered significant. See also Table S1 for detailed statistical information.

## Results

### 6 months old APP-GAD-Cre mice exhibited impaired NREM sleep, decreased delta power, and increased sleep fragmentation

**Fig. 1:**
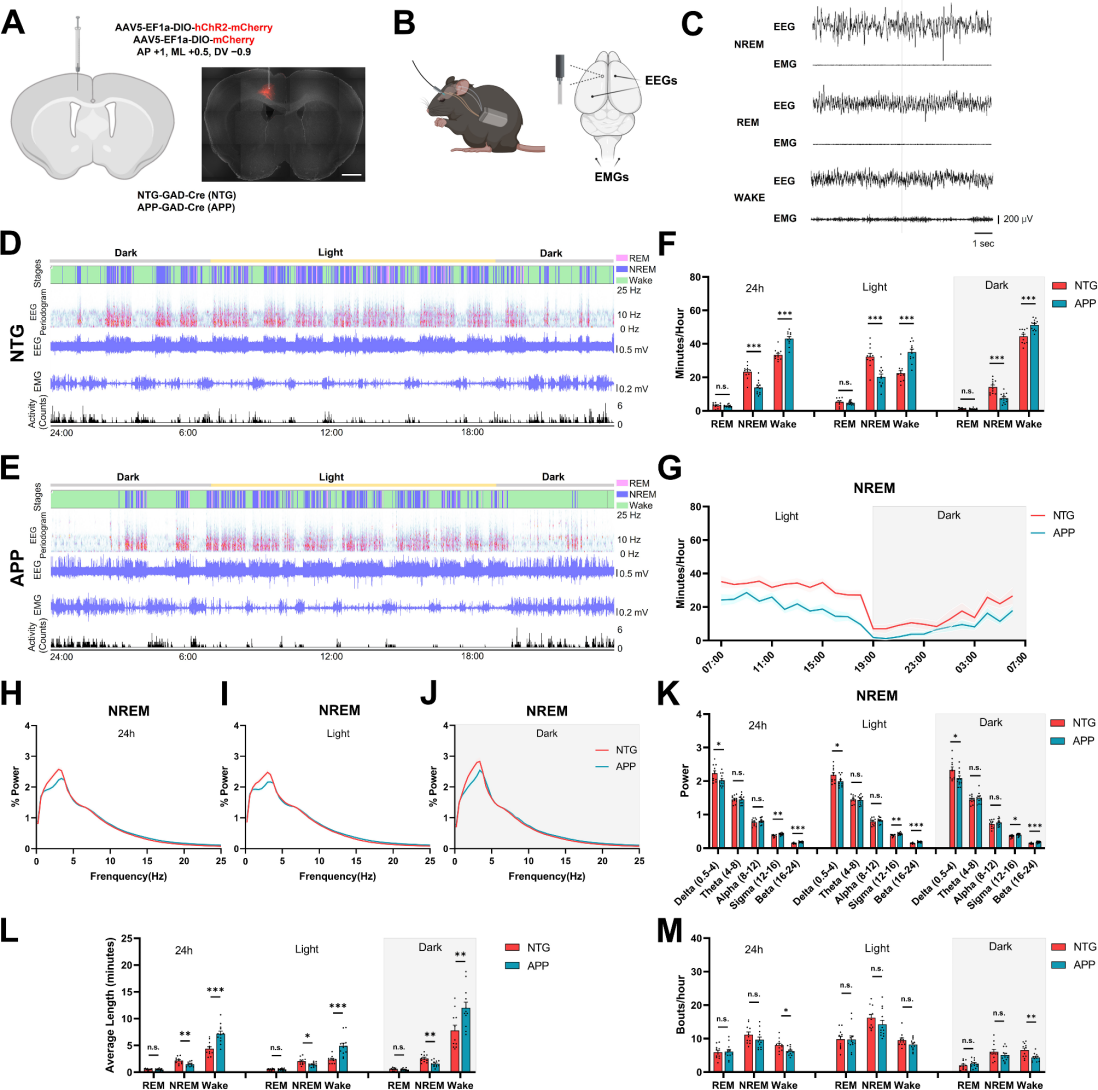
Impaired NREM sleep, decreased delta power, and increased sleep fragmentation in APP mice at 6 months of age. (**A**) Left, diagram showing viral injection strategy to target GABAergic neurons with mCherry or ChR2-mCherry. Right, representative photomicrograph showing GABAergic neurons expressing ChR2-mCherry with DAPI (Gray). The dashed line shows the approximate location of the cannula track. Scale bar, 1 mm. (**B**) Left, diagram showing placement of EEG/EMG implant. Right, placement of EEG, EMG electrodes, and fiber-optic cannula on the skull. EMG electrodes were placed within the nuchal musculature. (**C**) Representative EEG and EMG traces during NREM, REM and wake states. (**D** and **E**) Overall 24-hour sleep pattern and sleep architecture of the NTG (**D**) and APP (**E**) mice. (**F**) Averaged time spent in each sleep-wake cycle stage (NREM, REM and wake) during 24-hour, 12-hour dark phase and 12-hour light phase of NTG and APP mice. (**G**) Time course of the changes in NREM sleep in NTG and APP mice. (**H**-**J**) Relative power spectral density of NREM sleep during 24-hour, 12-hour llight phase and 12-hour dark phase of NTG and APP mice. (**K**) The average EEG power density in the delta (0.5–4 Hz), theta (4–8 Hz), alpha (8–12 Hz), sigma (12–16 Hz) and beta (16–24 Hz) bands during NREM sleep during 24-hour, 12-hour light phase and 12-hour dark phase of NTG and APP mice. (L and M) Average length (L) and bouts count (M) in each sleep-wake cycle stage (NREM, REM and wake) during 24-hour, 12-hour dark phase and 12-hour light phase of NTG and APP mice. All data are expressed as means ± standard error. The number of mice examined: NTG = 11 mice; APP = 12 mice. *P < 0.05, **P < 0.01, and ***P < 0.001. n.s. not significant

To investigate the role of the GABAergic interneurons in the context of AD, we generated a mouse model where the general GABAergic (glutamic acid decarboxylase, or GAD-expressing) neuronal population could be targeted in the presence of amyloidosis. We crossed heterozygous APP/NTG mice [13] with homozygous GAD-Cre animals [30], thus generating APP-GAD-Cre mice, and NTG-GAD-Cre mice that served as controls (Fig. 1A). Previous literature suggested that APP mice exhibited sleep disruptions early in the disease progression preceding the cognitive deficits and AD neuropathology [16]. Thus, we first examined whether sleep disruptions were present in 6-month-old APP-GAD-Cre mice when compared to NTG-GAD-Cre controls, using an EEG/EMG telemetry system (Fig. 1B). Mice exhibited NREM and REM sleep as well as awake states while maintained individually in home cages (Fig. 1C).

Analysis of sleep architectures revealed that both NTG-GAD-Cre (NTG) and APP-GAD-Cre (APP) mice were nocturnal and had a sleep pattern with the main sleep period identified during the light phase, while also spending a considerable amount of time sleeping during the dark phase. In general, APP mice exhibited disrupted sleep patterns, with the hypnogram showing interruptions in NREM sleep (Fig. 1D, E). APP mice spent more time awake and less time in NREM sleep during the 24-hour period (Fig. 1F). Furthermore, APP mice spent significantly more time awake and less time in NREM sleep during both light and dark periods, indicating poor sleep quality compared to that of NTG controls (Fig. 1F, G). We did not detect significant differences in time spent in REM sleep (Fig. 1F). We then performed Fourier transform analysis to determine the power-frequency relationships. APP mice exhibited lower delta power (0.5-4 Hz) during NREM sleep when assessed across the 24-hour period, during the light period or during the dark period. A decrease in delta power was accompanied by an increase in sigma and beta power in APP mice (Fig. 1H-K), suggesting hyperactivity within the circuit. Analyses of powers during REM sleep (Supplemental Fig. 11A-C, G) and wakefulness (Supplemental Fig. 1D-F, H), further confirmed a shift to higher frequencies, which is consistent with previous sleep research using AD mouse models [34]. Moreover, we discovered increased sleep fragmentation in APP mice with a significant reduction in NREM bout length and bout number comparable to that in NTG mice (Fig. 1L, M).

Overall, these results demonstrate that APP mice exhibit deficits in NREM sleep, erosion of delta power and decreases in individual bout length at 6 months of age. These data are consistent with increases in circuit hyperactivity evident due to increases in powers of higher frequency bands.

### Optogenetic stimulation of GABAergic interneurons ameliorated sleep deficits in APP-GAD-Cre mice

Previous studies reported that GABAergic interneurons are important for sleep generation and regulation, particularly for SWA during NREM sleep [35, 36]. Excessive Aβ induces neuronal dysfunction and downregulates the activity of GABAergic interneurons, which eventually leads to circuit failure in AD [37]. We previously reported deficits in inhibitory elements of the circuit, specifically decreases in GABA, GABA_A_ and GABA_B_ receptors in young APP mice. Furthermore, GABA administration rescued slow wave power in APP mice [14]. Thus, in this study, we determined whether optogenetic stimulation of cortical GABAergic interneurons at the slow wave frequency could rescue sleep deficits and restore NREM delta power in APP mice.

**Fig. 2:**
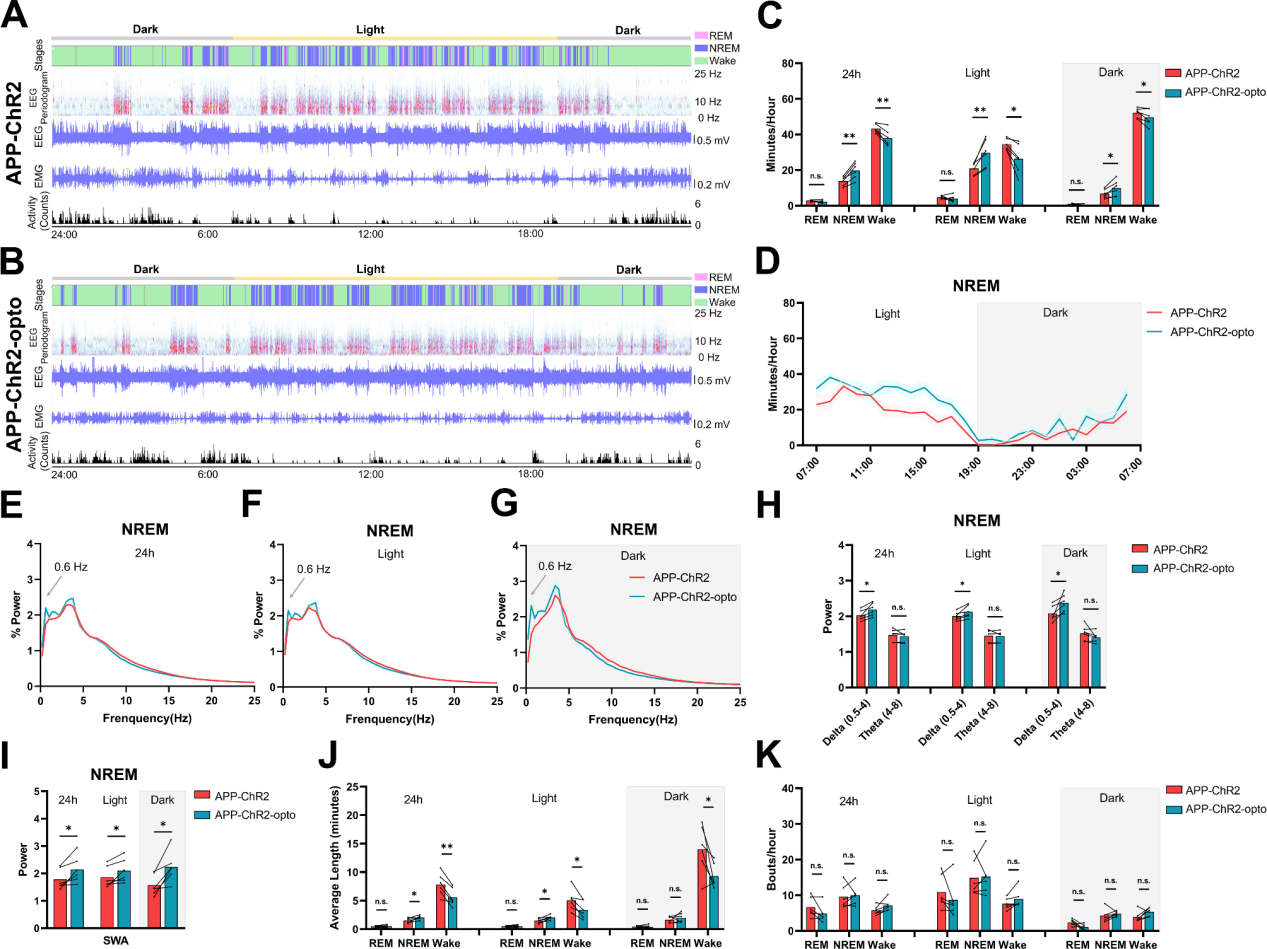
Optogenetic stimulation of GABAergic neurons increased SWA and delta power, rescued NREM sleep and promoted sleep integrity in APP mice. (**A** and **B**) Overall 24-hour sleep pattern and sleep architecture of the APP mice before (**A**) and during (**B**) stimulation on ChR2. (**C**) Averaged time spent in each sleep-wake cycle stage (NREM, REM and wake) during 24-hour, 12-hour dark phase and 12-hour light phase of APP mice before and during stimulation on ChR2. (**D**) Time course of the changes in NREM sleep in APP mice before (APP-ChR2) and during stimulation (APP-ChR2-opto) on ChR2. (**E**-**G**) Relative power spectral density of NREM sleep during 24-hour, 12-hour dark phase and 12-hour light phase of APP mice before and during stimulation on ChR2. (**H**) The average EEG power density in the delta (0.5–4 Hz) and theta (4–8 Hz) bands during NREM sleep during 24-hour, 12-hour dark phase and 12-hour light phase across conditions. (**I**) The average SWA (0.5–1 Hz) power during NREM sleep during 24-hour, 12-hour dark phase and 12-hour light phase across conditions. (**J** and **K**) Average length (**J**) and bouts count (**K**) in each sleep-wake cycle stage (NREM, REM and wake) during 24-hour, 12-hour dark phase and 12-hour light phase across conditions. All data are expressed as means ± standard error. The number of mice examined: APP = 6 mice/group. *P < 0.05, **P < 0.01. n.s. not significant

We used Cre/Lox recombination technology to express ChannelRhodopsin-2 (ChR2) exclusively in GABAergic interneurons in the anterior cortex of APP-GAD-Cre (APP) and NTG-GAD-Cre (NTG) mice (Fig. 1A). We optogenetically targeted interneurons in the anterior cortex because the anterior cortex is known to be the origin of endogenous slow oscillations [38]. APP mice exhibited sleep rescue during optogenetic stimulation of ChR2 (APP-ChR2-opto), compared to those prior to stimulation (APP-ChR2) (Fig. 2A, B). Optogenetic stimulation of GABAergic interneurons increased the amount of time APP mice spent in NREM sleep. This phenomenon was observed in both light and dark phases (Fig. 2C, D). APP mice spent less time awake during stimulation. REM sleep was not affected significantly by stimulation (Fig. 2C). Fourier transform analysis revealed an increase in delta power (0.5-4 Hz) during stimulation compared to baseline (Fig. 2E-H). SWA (0.5-1 Hz), the major restorative feature of NREM sleep, was significantly improved during stimulation across the 24-hour period, during light and dark periods (Fig. 2I). Additionally, optogenetic stimulation ameliorated sleep fragmentation as indicated by prolonged NREM bout lengths particularly in the light phase (Fig. 2J), while maintaining the bout numbers (Fig. 2K).

In contrast, light stimulation of mCherry in absence of ChR2 failed to significantly affect sleep architectures in both NTG (Supplemental Fig. 2) and APP mice (Supplemental Fig. 3). No significant differences in NREM, REM sleep and wake duration were found in mCherry groups during light and dark phases (Supplemental Fig. 2A, B; Supplemental Fig. 3A, B). In addition, Fourier transform analysis revealed that the power spectra density plots were comparable (Supplemental Fig. 2C-G; Supplemental Fig. 3C-G). Similarly, there were no significant differences in sleep/wake durations and power density between APP-mCherry or APP-ChR2 groups in absence of light stimulation (Supplemental Fig. 4A-C). Thus, light stimulation of ChR2 resulting in optogenetic activation of GABAergic interneurons was necessary for NREM sleep rescue.

Altogether our data suggest that optogenetic stimulation of GABAergic interneurons rescued sleep deficits, and improved delta power as well as SWA in APP mice.

### Chronic optogenetic stimulation of GABAergic interneurons reduced amyloid plaque deposition in APP mice

**Fig. 3:**
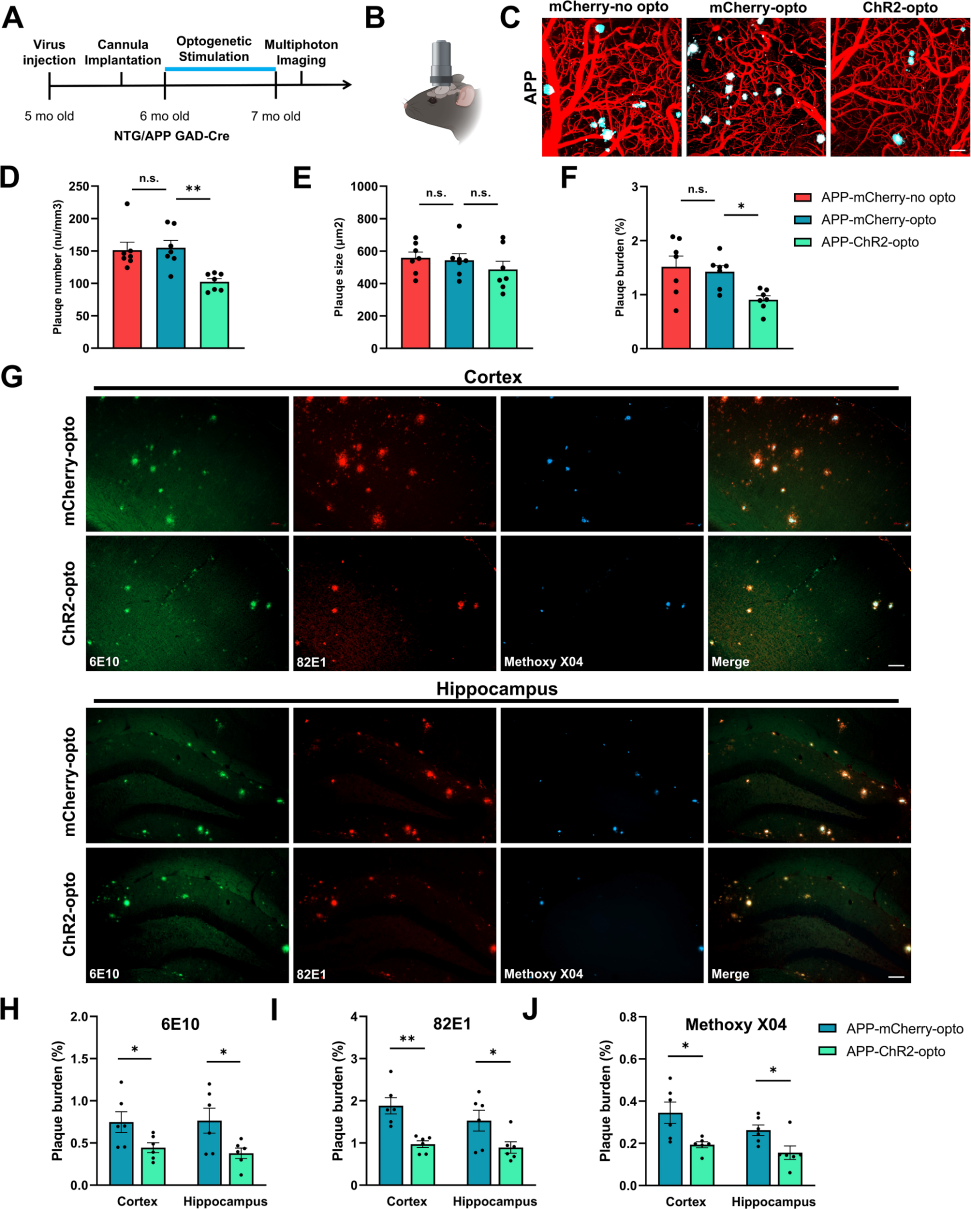
Effect of chronic optogenetic stimulation of GABAergic neurons on amyloid plaque deposition in APP mice. (**A** and **B**) Experimental design. After AAV infusion and cannula installation, mice received 1-month of continuous optogenetic stimulation. Multiphoton imaging (**B**) was performed after treatment. (**C**) Representative multiphoton images of Methoxy-X04 positive amyloid plaques (Cyan) and blood vessels (Red) in APP mice in absence of stimulation (APP-mCherry-no opto), in presence of stimulation of mCherry (APP-mCherry-opto) and during stimulation of ChR2 (APP-ChR2-opto). (**D**) Amyloid plaque number across conditions. (**E**) Amyloid plaque size across conditions. (**F**) Amyloid plaque burden across conditions. (**G**) Representative images of 6E10, 82E1 and Methoxy-X04 positive amyloid plaques in cortex and hippocampus within postmortem sections. (**H**-**J**) 6E10 (H), 82E1 (**I**) and Methoxy-X04 (**J**) positive amyloid plaque burden across conditions. All data are expressed as means ± standard error. The number of mice examined: n = 6–7 mice/group. *P < 0.05, **P < 0.01. n.s. not significant. Scale bars: 50 μm

Since sleep disruptions were shown to facilitate Aβ accumulations [39, 40], we next determined whether restoring sleep deficits could slow Aβ deposition. To achieve this, 6-month-old APP mice were treated with chronic optogenetic stimulation of ChR2 (ChR2-opto) targeted to cortical GABAergic neurons. Since APP mice showed deficits in NREM sleep during the day and night, Optogenetic treatment was performed continuously for 4 weeks. After treatment, a cranial window was installed over the right posterior cortex, contralateral to the stimulation site (Fig. 3A, B). Since slow oscillations activated in the anterior cortex propagate to the contralateral side, we chose to stimulate ChR2 in the anterior left cortex and image amyloid deposition in the posterior right. That way, we were able to avoid the confound of imaging the neurons that were directly activated by optogenetics. Amyloid plaques were labeled with Methoxy-X04 and monitored using multiphoton microscopy in anesthetized mice (Fig. 3C).

Amyloid plaque number was significantly lower after optogenetic stimulation in the ChR2-opto group when compared to other conditions in APP mice (Fig. 3D). Plaque size was comparable in all three groups (Fig. 3E). Amyloid plaque burden, which considers the number and size of plaques, was also significantly lower in optogenetically treated APP mice (Fig. 3F). These data demonstrated that chronic optogenetic stimulation of GABAergic neurons resulted in lower amyloid deposition in APP mice compared to APP-mCherry-opto control mice. Moreover, amyloid burden did not differ significantly between APP-mCherry-opto and APP-mCherry-no opto groups, demonstrating that the blue light used here did not result in detectable toxicity (Fig. 3D-F).

Since multiphoton microscopy allowed monitoring amyloid under the cranial window in a small cortical region, we verified amyloid plaque data in a greater cortical region as well as hippocampus, a deeper brain region inaccessible by multiphoton microscopy. We performed immunostaining on post-mortem brain tissue of the same APP mice chronically treated with optogenetic activation of ChR2 or mCherry as described above. Brain sections were immunostained with anti-amyloid β antibody 6E10 reactive to amino acid residue 1–16 of amyloid β, and 82E1 which recognized the N-terminus of Aβ but not full-length APP. Immunostaining was compared with Methoxy-X04. While Methoxy-X04 labeled dense cores of amyloid plaques, 6E10 and 82E1 decorated the periphery as well as the cores (Fig. 3G). Chronic optogenetic stimulation resulted in lower amyloid plaque burden both in the cortex and the hippocampus. 6E10 and 82E1 immunoreactivity revealed that amyloid plaque burden was significantly lower as a result of chronic optogenetic stimulation in the ChR2 group compared to the control mCherry group (Fig. 3G-I). Similarly, the Methoxy-X04 data revealed lower plaque burden after chronic optogenetic stimulation (Fig. 3G, J). These post-mortem data were all consistent with our in vivo findings using multiphoton microscopy. Finally, APP mice expressing ChR2 in absence of optogenetic stimulation (ChR2-no opto) failed to show lower plaque deposition (Supplemental Fig. 5A-C).

Taken together, chronic optogenetic stimulation of GABAergic neurons ameliorated amyloid plaque deposition in APP mice.

### Chronic optogenetic stimulation of GABAergic interneurons improved neuronal calcium homeostasis in APP mice

**Fig. 4:**
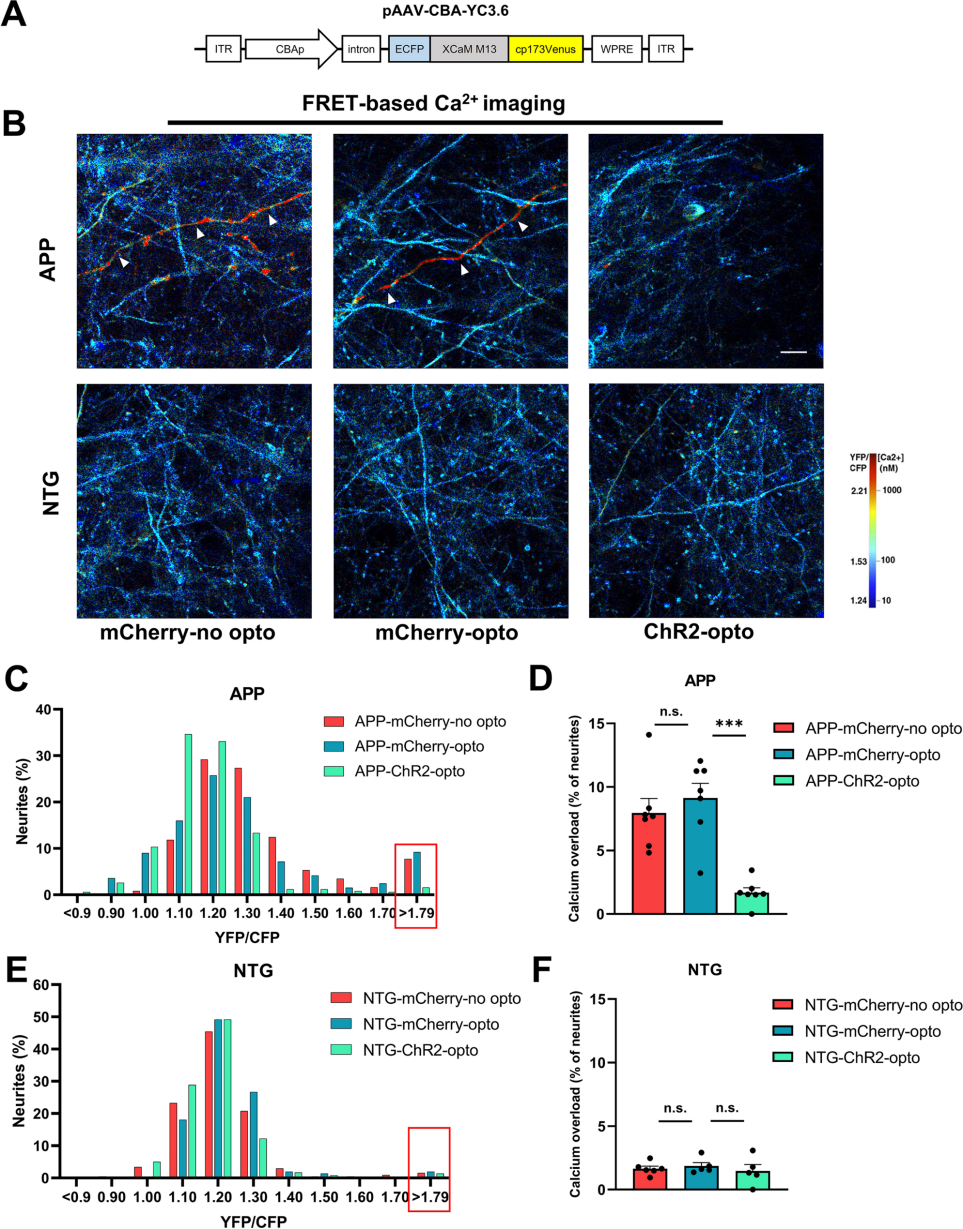
Effect of chronic optogenetic stimulation of GABAergic neurons on Calcium overload in NTG and APP mice. (**A**) Diagram of AAV-CBA-YC3.6 construct. (**B**) Representative multiphoton images pseudocolored according to the intraneuronal calcium concentration in NTG and APP mice in absence of optogenetic stimulation (mCherry-no opto), in presence of optogenetic stimulation of mCherry (mCherry-opto) and optogenetic stimulation of ChR2 (ChR2-opto). Neuronal processes exhibiting calcium overload are shown in red (see arrowheads). (**C**) Histogram showing the distribution of YFP/CFP ratios in neurites expressing YC 3.6 of APP mice. (**D**) The percentage of neurites exhibiting calcium overload across conditions in APP mice. (**E**) Histogram showing the distribution of YFP/CFP ratios in neurites expressing YC 3.6 of NTG mice. (**F**) The percentage of neurites exhibiting calcium overload across conditions in NTG mice. All data are expressed as means ± standard error. Neuronal calcium overload was defined as a YFP/CFP ratio larger than 2 standard deviations above the average YFP/CFP ratio in the neurons of NTG mice. The ratio of YFP/CFP > 1.73 was considered calcium overload. The number of mice examined: n = 5–7 mice/group. ***P < 0.001. n.s. not significant. Scale bar: 50 μm

In addition to depositing amyloid plaques, APP mice contain a small cortical neuronal population that is vulnerable to amyloid β-dependent calcium dysregulation resulting in calcium elevations [31]. This results in calcium overload within neuronal processes, neurites [41]. To determine whether chronic optogenetic stimulation of GABAergic neurons improved neuronal calcium homeostasis, we expressed the radiometric calcium sensor, Yellow Cameleon 3.6 (YC3.6), in the cortex of APP mice and NTG controls (Fig. 3A A, B). YC3.6 is a genetically encoded calcium sensor containing YFP and CFP [42]. A YFP/CFP ratio greater than two standard deviations above the NTG mean, 1.79, constituted calcium overload (Fig. 4C, red box, D). This value translated into an intracellular calcium concentration greater than 235nM [31]. Neurites exhibiting calcium overload are shown (Fig. 4B, red neurites, white arrowheads). Therefore, restoration of neuronal calcium levels would serve as a functional indicator of treatment efficacy [43].

As anticipated, APP mice expressing mCherry contained more neurites with calcium overload compared to healthy NTG controls (Fig. 4B, top left and middle panels, red neurites, white arrowheads, Fig. 4B bottom left and middle panels). Optogenetic treatment of ChR2 led to a lower percentage of neurites with calcium overload in APP mice (Fig. 4B, upper right panel). Histogram analysis revealed the presence of a small yet vulnerable neuronal population with calcium elevations, exhibiting YFP/CFP ratio of > 1.79 in APP mice with mCherry (Fig. 4C, red box). Compared to mCherry group, optogenetic stimulation of ChR2-expressing GABAergic interneurons resulted in a lower percentage of neurites with calcium overload (Fig. 4C, D). To control blue light toxicity, we examined APP mice expressing mCherry with and without light treatment. APP-mCherry-opto mice exhibited neuronal calcium overload comparable to APP-mCherry-no opto mice, demonstrating that blue light had no major toxic impact on neuronal calcium homeostasis in APP mice (Fig. 4B-D).

In addition, optogenetic stimulation of ChR2-expressing GABAergic interneurons in healthy NTG mice did not significantly alter the percentage of neurites with calcium overload compared to the NTG-mCherry-opto group (Fig. 4B, E, F). Thus, optogenetic stimulation of GABAergic interneurons had no significant impact on calcium homeostasis in healthy NTG mice. Similarly, NTG-mCherry-opto and NTG-mCherry-no opto groups had a comparable percentage of neurites with calcium overload (Fig. 4B, E, F).

Thus, chronic optogenetic stimulation of GABAergic neurons decreased neuronal calcium overload and restored neuronal calcium homeostasis in APP mice.

### Chronic optogenetic stimulation of GABAergic interneurons improved memory consolidation in APP mice

**Fig. 5:**
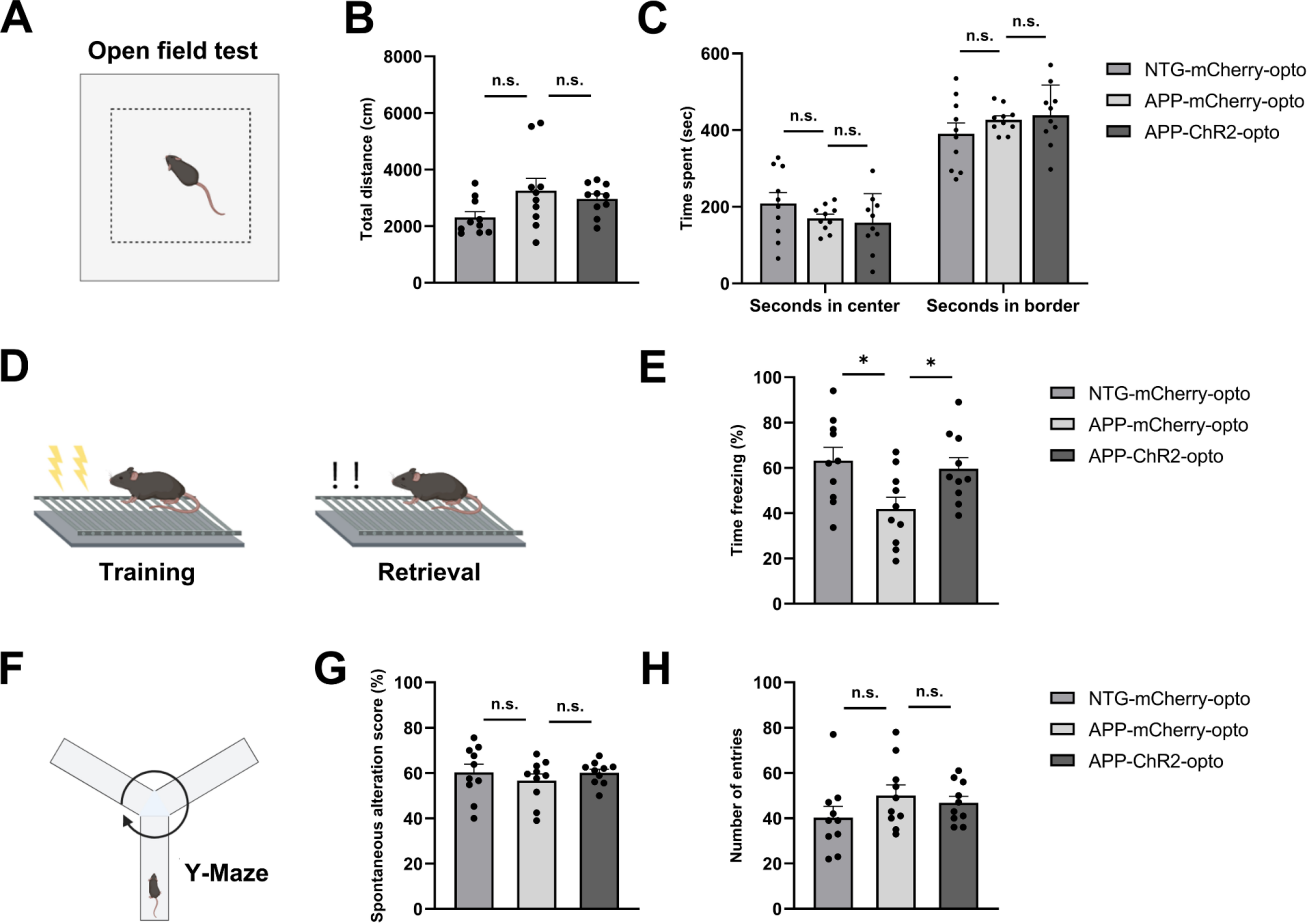
Chronic optogenetic stimulation of GABAergic neurons improved memory performance in APP mice. (**A**) Schematic diagram of the open field test. (**B**) The total distance traveled during open field test across conditions. (**C**) Time spent exploring the center or border zones. (**D**) Schematic diagram of fear conditioning test. (**E**) Percentage of time spent freezing during fear recall. (**F**) Schematic diagram of the Y-maze spontaneous alternation is shown. (**G**) Percentage of spontaneous alternation (% alternation) activities across conditions. (**H**) The number of total entries. All data are expressed as means ± standard error. The number of mice examined: n = 10 mice/group. *P < 0.05. n.s. not significant

We next assessed whether optogenetic stimulation of GABAergic neurons affected memory. We subjected a different cohort of NTG and APP mice to a battery of behavioral tests after 2 weeks of optogenetic treatment of ChR2 or light treatment of mCherry. The mice were first tested in an open field to determine whether optogenetic stimulation altered the locomotor activity or induced any anxiety-like behaviors (Fig. 5A). The total distance traveled was comparable across conditions (Fig. 5B). Time spent in the center was similar across conditions, as was time spent in the border of open field (Fig. 5C). Thus, APP mice did not exhibit any locomotor impairments, nor anxiety-like behaviors.

We performed contextual fear conditioning to test the hippocampus-dependent associative emotional memory in the animals [44]. The APP mice were reported to exhibit cognitive deficits in contextual memory as early as 4–6 months of age [45]. On the first day, animals were subjected to electric foot shocks. After the fear acquisition, animals were allowed to sleep. The next day, their sleep-dependent memory consolidation was tested in the same chamber yet without foot shocks (Fig. 5D). The percentage of time spent freezing was used as an index of fear memory. As expected, the APP-mCherry-opto mice showed impairments in memory consolidation with less time spent freezing compared to the NTG-mCherry-opto group (Fig. 5E). Interestingly, the freezing levels were significantly higher in APP-ChR2-opto group, indicating that optogenetic stimulation of GABAergic neurons restored memory consolidation in APP mice (Fig. 5E).

The amygdala is required for the acquisition and expression of learned fear responses in mice, and the hippocampus-amygdala circuit is engaged during contextual fear conditioning tests [44, 46]. Therefore, we examined plaque deposition in the amygdala after treatment. Optogenetic stimulation of GABAergic neurons failed to reduce plaque deposition in the amygdala of APP mice (Supplemental Fig. 6A-D). This might be explained by the fact that the amygdala had lower plaque load compared to the cortex and hippocampus during the ages tested. In addition, we tested the animals in the Y-maze test (Fig. 5F) to measure spatial working memory, as a spontaneous alternation score [47]. We observed no impairments in APP mice at this age. Chronic optogenetic treatment did not alter the score significantly (Fig. 5G, H).

Overall, these data demonstrate that optogenetic stimulation of GABAergic interneurons rescued sleep-dependent memory consolidation in APP mice without altering sleep-independent working memory. Furthermore, optogenetic treatment did not significantly affect the locomotion or anxiety levels of the APP mice.

### Chronic optogenetic stimulation of GABAergic interneurons induced alterations of microglial morphological features and phagocytic ability

**Fig. 6:**
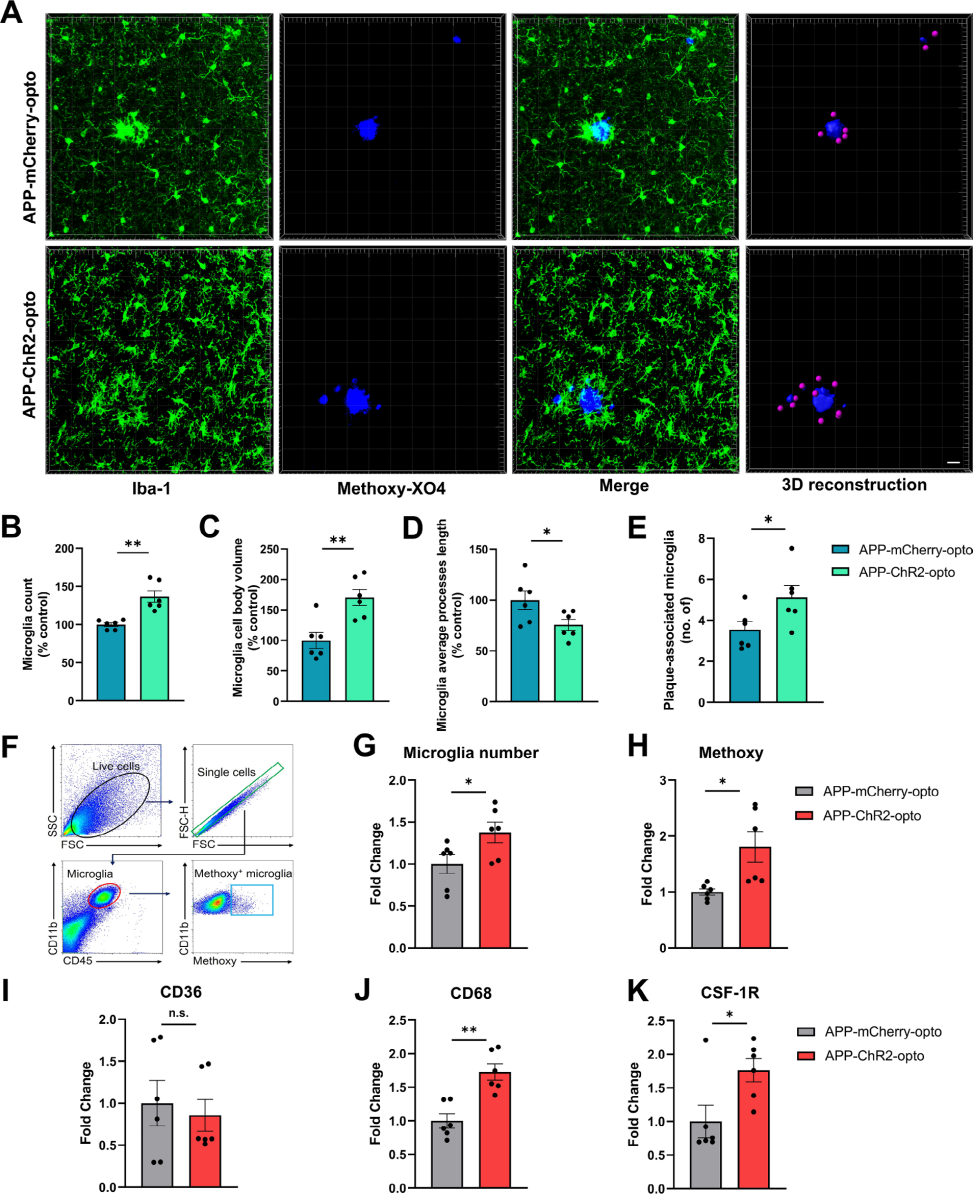
Chronic optogenetic stimulation of GABAergic neurons altered microglia number and morphology, upregulated expression of phagocytic markers and enhanced phagocytic activity in APP mice. (**A**) Representative confocal Z-projections depicting Iba-1 positive microglia (Green) and Methoxy-XO4 positive plaques (Blue). (**B**-**E**) Microglia number (**B**), microglia cell body volume (**C**), microglia process length (**D**) and the number of plaque associated microglia (**E**). (**F**) Gating strategy used to identify CD11b + CD45lo microglia. The proportion of Aβ-phagocytic microglia (MeX04 + microglia). (**G**) Quantitation of the microglial cell population. (**H**) Quantification of the Methoxy-X04^+^CD11b^+^CD45^low^ microglia. (I-K) Quantitation of microglial CD36 (**I**), CD68 (**J**) and CSF-1R (**K**) expression. All data are expressed as means ± standard error. The number of mice examined: n = 6 mice/group. *P < 0.05 and **P < 0.01. n.s. not significant. Scale bars: 50 μm

Accumulating evidence suggests that microglia are regulated by sleep and play a role in AD pathology [25, 27, 29]. Our data demonstrated that sleep deficits were rescued by optogenetic stimulation of GABAergic neurons in APP mice. Thus, we investigated the effect of 2-week long optogenetic treatment on microglia. First, we immunostained the post-mortem brain sections of chronically treated animals with antibodies against the microglial marker Iba1. Amyloid plaques were labeled with Methoxy-XO4. Images were acquired using a confocal microscope with a 40X objective at identical settings (Fig. 6A). The number of reactive microglia was significantly elevated in mice in APP-ChR2-opto condition compared to those in APP-mCherry-opto condition (Fig. 6B). Increases in cell body size and decreases in process length were observed indicating a shift towards a phagocytic state as a result of optogenetic treatment (Fig. 6C, D). Additionally, microglia-Aβ clustering analyses were performed to evaluate the microglial clustering patterns around amyloid plaques (Fig. 6A). Using three-dimensional images of each amyloid deposit, the number of microglia located within a 25 μm radius of the deposit was quantified using Imaris. Optogenetic treatment increased the number of reactive microglia surrounding amyloid plaques (Fig. 6A, E). Overall, our data showed that optogenetic stimulation resulted in unique morphological changes within microglia.

To further assess microglial function, we performed flow cytometry on cells isolated from fresh tissue and identified microglia with CD11b and CD45 markers (Fig. 6F). We saw an increase in microglia number as a result of optogenetic treatment consistent with immunohistochemical data (Fig. 6G, B). To investigate the phagocytic ability of microglia, the percentage of viable Methoxy-X04^+^CD11b^+^CD45^low^ microglia as well as phagocytic markers CD36 and CD68 expression was measured. Optogenetic treatment resulted in an increased percentage of Methoxy-X04^+^CD11b^+^CD45^low^ microglia (Fig. 6H) as well as in elevated expression of CD68 (Fig. 6J) compared to the mCherry group. This indicated increased Aβ clearance ability by microglia in the APP-ChR2-opto group. In addition, optogenetic treatment increased the expression of the microglia proliferation marker CSF-1R (Fig. 6K), consistent with the increased number of microglia observed. Moreover, APP mice expressing ChR2 without optogenetic stimulation (ChR2-no opto) showed no significant difference in microglia number or phagocytic ability (Supplemental Fig. 5D, E).

Taken together, these results demonstrate that optogenetic stimulation of ChR2 targeted to GABAergic neurons increased reactive microglial proliferation, induced a clustering phenotype around plaques, and promoted Aβ phagocytosis by microglia that resulted in clearance of Aβ.

**Chronic optogenetic stimulation of GABAergic interneurons combined with sleep deprivation as well as 40 Hz stimulation failed to result in microglia-dependent amyloid plaque clearance in APP mice**.

Increasing evidence suggests that sleep disruption may also play a role in AD pathogenesis [1, 2, 15, 48]. Since optogenetic stimulation of GABAergic neurons improved sleep and slowed AD progression, it was important to determine whether sleep restoration was necessary. Thus, we tested whether sleep restoration resulting from optogenetic activation of interneurons led to slowing of AD progression in APP mice. Sleep deprivation is known to reduce total sleep time in mice [33]. APP mice were treated with optogenetic activation of ChR2 while being sleep deprived for 6 h each day (APP-ChR2-opto-SD). Their sleep patterns were compared to their baseline levels prior to optogenetic stimulation and sleep deprivation in the same mice (APP-ChR2). Interestingly, sleep deprivation ablated previously observed improvements in NREM sleep associated with optogenetic treatment (Supplemental Fig. 7A, Fig. 2C). We further examined the effect of sleep deprivation in the presence of stimulation on plaque deposition and microglia numbers. Amyloid plaque burden remained high in the cortex and the hippocampus of APP-ChR2-opto-SD mice (Supplemental Fig. 7B-D). Moreover, there was no significant difference in microglia number or its Aβ phagocytic ability in the APP-ChR2-opto-SD group compared to the control (Supplemental Fig. 7E, F).

Moreover, optogenetic stimulation of cortical interneurons at 40 Hz failed to significantly alter amyloid plaque deposition and microglia numbers in APP mice compared to non-treated APP mice (Supplemental Fig. 8).

Therefore, the frequency of optogenetic stimulation at 0.6 Hz was critical to elicit the protective effects of optogenetic stimulation of GABAergic neurons on plaque deposition and microglia through sleep restoration in APP mice.

## Discussion

The amyloid cascade hypothesis has been well-established as a prominent pathological pathway of amyloid in AD. Increasing evidence suggests a bidirectional relationship between Aβ and sleep [1, 2, 11]. Sleep disruptions have been reported as part of AD progression and could contribute to AD pathogenesis at preclinical stages. The exacerbation of AD pathology could further disrupt sleep by fragmenting it, decreasing NREM sleep and SWA as well as increasing time spent awake [3, 4, 10, 12]. Alternatively, increasing NREM sleep and SWA may be beneficial in slowing AD progression.

Here, use of an EEG/EMG telemetry system revealed sleep disruptions in APP mice at 6 months of age. APP mice spent more time awake and less time in NREM sleep. They also exhibited lower delta power and increased sleep fragmentation. These results are consistent with the observations reported previously by other investigators. APP mice were shown to exhibit impairments in NREM sleep and delta power at 5 months of age prior to plaque deposition [49]. In another study, 3-month-old APP mice exhibited significant deficits in NREM sleep preceding the cognitive deficits and AD neuropathology [16].

A recent study established a causal relationship between GABAergic interneuron function and sleep regulation. Interneurons control wakefulness by suppressing dopaminergic drive. Chemogenetic activation of GABAergic interneuron induced NREM sleep while suppressing wakefulness in NTG mice [50]. Thus, we targeted GABAergic interneurons optogenetically. Optogenetic stimulation of GABAergic interneurons rescued sleep disruptions and sleep fragmentation by improving NREM sleep, delta power and SWA in APP mice. Thus, we were able to rescue sleep deficits in a mouse model of AD. We next explored the extent to which optogenetic targeting of interneurons could slow AD pathophysiology.

An increasing number of studies suggests that sleep deficits lead to AD [1, 2, 51]. Aβ fluctuates diurnally within the brain interstitial fluid as well as within the cerebrospinal fluid. Soluble Aβ levels are higher during wakefulness and lower during sleep [52]. Sleep is associated with an increase in the exchange of cerebrospinal fluid with interstitial fluid, which leads to an increased rate of β-amyloid clearance [53]. The role of sleep in amyloid pathogenesis has been further assessed with sleep deprivation studies in humans and animal models. Acute sleep deprivation increased soluble Aβ in human CSF by 25–30% via increased overnight Aβ production relative to sleeping controls [39]. Both Tg2576 mice and APP mice exposed to chronic sleep deprivation for 20 h daily over a 21-day period had significant increases in amyloid plaque pathology. The increased Aβ levels significantly correlated with wake times [40]. Aβ burden in the medial prefrontal cortex correlated significantly with the severity of impairments in NREM sleep and SWA [3]. APP mice overexpress mutant human APP that results in the generation of human Aβ, which aggregates as oligomers, and gets deposited in form of amyloid plaques [54]. Remarkably, chronic optogenetic stimulation of GABAergic neurons was sufficient to slow Aβ accumulation in APP mice, suggesting that the GABAergic interneuron dysfunction actively contributes to AD progression. Calcium is critical to the maintenance of proper neuronal function [55]. Thus, calcium homeostasis is tightly regulated within neurons. Calcium homeostasis is disrupted in AD since Aβ leads to neuronal calcium elevations [31]. A higher proportion of neurites exhibited elevated levels of calcium (calcium overload) in APP mice compared to NTG controls. Here we show that optogenetic stimulation of GABAergic neurons restored calcium homeostasis, evidenced by decreases in the number of neurites exhibiting calcium overload.

Cognitive decline is a problematic and disabling consequence of AD, with impairments in hippocampus-dependent memory being one of the most debilitating symptoms [3, 56]. A change in sleep architecture is pronounced in patients with mild cognitive impairment, which is often a prodromal phase of dementia, and those with dementia caused by AD [1, 2]. Aβ accumulation could be correlated significantly with sleep disruption as evidenced by impaired NREM sleep and SWA, leading to memory decline [3]. Experimentally increasing SWA during NREM sleep, specifically in the slow, < 1 Hz frequency range, caused an enhancement of memory consolidation and thus long-term memory retention in young adults [57]. Our results show impairments in contextual fear memory in 6-month-old APP mice, consistent with an earlier study using the same animal model [45]. A major finding from our study is the improvement of sleep-dependent memory consolidation after optogenetic stimulation of GABAergic neurons. Interestingly, working memory tested in the Y-maze was not significantly altered. It is possible that the APP mice used in our experiment were too young to exhibit deficits in spatial working memory. To gain a deeper understanding of sleep restoration on memory as well as their relationship with the hippocampus, additional behavior tests such as the Morris water maze and novel object recognition are warranted in future studies.

In this study, optogenetic stimulation of GABAergic neurons resulted in the proliferation of microglia and induced profound morphological changes. Specifically, it transformed microglia toward a phagocytic state. An increased microglial clearance ability was further verified through examination of phagocytosed Aβ and expression of CD68. To further determine the role of sleep restoration in mediating the beneficial effects of optogenetic stimulation of GABAergic interneurons, we conducted a chronic sleep deprivation experiment in combination with optogenetic stimulation. When properly executed, sleep deprivation is known to reduce sleep effectively [57]. Indeed, we determined that optogenetic stimulation of GABAergic neurons failed to rescue sleep deficits when animals were sleep deprived for 6 h each day. Most importantly, sleep deprivation prevented plaque clearance by microglia. This demonstrates that sleep restoration is responsible for the protective effects of optogenetic stimulation of GABAergic neurons on plaque deposition and microglia regulation in APP mice. The immune-supportive function of sleep occurs primarily during NREM sleep [23]. In human studies, boosting SWA during NREM sleep in men using auditory closed-loop stimulation improved endocrine activity, supporting peripheral immunity [24]. However, the regulatory effects of NREM sleep and SWA on innate immunity within the central nervous system were underinvestigated [2, 51]. Recent studies reported that sleep loss affected microglial morphology, phagocytosis, and Aβ clearance [28, 29]. This finding opened a possibility of influencing microglia, the primary innate immune cells of the brain, by boosting SWA and NREM sleep.

Microglia can detect and respond to neuronal activity in various conditions [58]. Neuronal hyperactivity during seizures, for example, can lead to the extension of microglial processes toward neurons in multiple brain regions [59]. Thus, the mechanisms underlying sleep and microglial function may also be related to how microglia sense neuronal activity [58]. Microglial responses to neuronal activity occur through distinct signaling pathways, including P2Y12 receptors on microglia responding to neuronal release of ADP [58]. Future studies will be needed to elucidate the exact mechanisms at play. In addition, a recent study demonstrated microglia can directly respond to GABA release, resulting in selective sculpting of developing inhibitory circuits [60]. Here, we speculate that optogenetic stimulation leads to a reprogramming of microglia towards activated states such as disease-associated microglia, which act as universal immune sensors of neurodegeneration [61, 62]. This postulation in particular merits deeper investigation using single-cell RNA sequencing. Detailed investigations are needed to identify the molecular and cellular mechanisms of how sleep affects microglia in the context of AD. At the same time, the potential contribution of other clearance mechanisms regulated by sleep, including the glymphatic pathway needs to be investigated in the future [1, 11, 15].

An earlier study showed that 40 Hz optogenetic stimulation of hippocampal parvalbumin-positive fast-spiking interneurons elicited gamma-frequency rhythmicity and reduced Aβ levels with a concomitant microglia response in 5XFAD mice [63]. We determined that 40 Hz optogenetic stimulation of a broad population of cortical interneurons failed to reduce amyloid plaque load in the cortex. This could be due to the use of distinct mouse models of amyloidosis: APP vs. 5XFAD mice. Furthermore, we targeted a broader population of interneurons compared to a parvalbumin-positive population [63]. Also, we targeted a different circuit in a distinct brain region, cortico-thalamic circuit in the cortex responsible for sleep, with slow oscillations at 0.6 Hz as a prevalent brain rhythm. The other study targeted gamma rhythm at 40 Hz in the hippocampus that is prevalent during wake behavior. Thus, it is critical to use the frequency of stimulation that is pertinent for the circuit, which activity is being restored.

Both studies targeted inhibitory interneurons in the context of AD. Each demonstrated that boosting these respective brain rhythms slowed AD progression. Collectively, these studies provide a strong rationale for the development of non-invasive stimulation strategies to slow AD progression. The effects of both studies found on microglia could be related to the crosstalk between neuronal activity and microglia as mentioned above.

One of the limitations of our current study is the use of isoflurane anesthesia. Recent studies found that anesthetics affect microglial dynamics, including process motility and territory surveillance [64, 65]. Microglial surveillance and injury responses are reduced in awake compared to anesthetized mice, likely due to reduced neuronal activity under anesthesia [64, 65]. Although we used anesthetics consistently across all the groups and thus the effect of isoflurane on the microglial dynamics would be omnipresent, future studies using unanesthetized mice are needed.

Taken together, we demonstrated that GABAergic interneurons play a critical role in sleep disruptions underlying AD. Optogenetic stimulation of inhibitory interneurons improved SWA and ameliorated sleep disruptions. It reduced amyloid plaque load and restored calcium homeostasis in neurons. It also improved sleep-dependent memory consolidation. Furthermore, optogenetic treatment resulted in microglial proliferation and morphological transformation of microglia towards phagocytic state, thus potentiating Aβ clearance. Our results provide strong evidence that specific targeting of GABAergic interneurons could ameliorate sleep disruptions and slow AD progression in an AD mouse model. This research could open the possibility of translating stimulation technologies to humans using sensory stimulation, such as acoustic stimulation, or transcranial Direct Current Stimulation.

## Conclusions

AD is a progressive neurodegenerative disorder that results in memory and cognitive impairments. Sleep disruptions, especially reductions of NREM sleep and SWA, may drive the development of AD neuropathology by increasing Aβ production and promoting a dysregulated state of microglia. Here, we provide evidence of sleep disturbances including decreased time spent in NREM sleep, decreased delta power, and increased sleep fragmentation in APP mice at 6 months of age. Optogenetic stimulation of GABAergic interneurons in the anterior cortex rescued sleep deficits and improved delta power as well as SWA. This provided a link between the restoration of inhibitory tone by activation of the general GABAergic interneuron population and the restoration of sleep in AD. Moreover, our results also indicated that chronic optogenetic stimulation of GABAergic interneurons slowed AD progression by reducing amyloid deposition, normalizing neuronal calcium homeostasis, and improving memory function. Additionally, we investigated the responses of microglia to sleep rescue from morphological and functional aspects. Optogenetic stimulation of GABAergic neurons increased microglial numbers, induced a clustering phenotype around plaques, and promoted Aβ phagocytosis by microglia that resulted in clearance of Aβ. Our study provided an understanding of sleep restoration by stimulating inhibitory, GABAergic interneurons in AD. It also provided new insights into the relationship between sleep and microglia-mediated innate immune response in AD pathogenesis. We suggest that targeting GABAergic interneurons might be important in developing treatments for AD.

### Electronic supplementary material

Below is the link to the electronic supplementary material.

**Supplementary Material 1**: Table S1. Supplemental Statistical Analysis

**Supplementary Material 2**: Supplemental Figures

## Data Availability

All data supporting the conclusions of this article are included within the article and in additional files provided.

## List of abbreviations

AAV: Adeno-Associated Virus
Aβ: Amyloidβ
AD: Alzheimer’s disease
APP: APPswe/PS1dE9
ChR2: ChannelRhodopsin-2
CNS: Central nervous system
CSF1R: Colony-stimulating factor 1 receptor
EEG: Electroencephalography
EMG: Electromyography
FRET: Fluorescence Resonance Energy Transfer
GABA: Gamma-aminobutyric acid
IACUC: Institutional Animal Care and Use Committee
ISF: Interstitial fluid
NREM: Non-rapid eye movement
NTG: Non-transgenic littermates
REM: Rapid eye movement
ROI: Regions of interest
SD: Sleep deprivation
SWA: Slow-wave activity
YC3.6: Yellow Cameleon 3.6
