# Supplementary material for: Sleep restoration by optogenetic targeting of GABAergic neurons reprograms microglia and ameliorates pathological phenotypes in an Alzheimer’s disease model
Source: Mol Neurodegener. 2023 Dec 1;18:93. doi: 10.1186/s13024-023-00682-9 (PMC10693059; doi:10.1186/s13024-023-00682-9)

**Supplemental Fig. 1. Power spectral density during REM sleep and wakefulness in APP and NTG mice at 6 months of age**.

(A-F) Relative power spectral density of REM sleep and wakefulness during 24-hour, 12-hour dark phase and 12-hour light phase of APP and NTG mice at 6 months of age.

(G and H) The average EEG power density in the delta (0.5–4 Hz), theta (4–8 Hz), alpha (8–12 Hz), sigma (12–16 Hz) and beta (16–24 Hz) bands in REM sleep and wakefulness during 24-hour, 12-hour dark phase and 12-hour light phase of APP and NTG mice at 6 months of age.

All data are expressed as means ± standard error. The number of mice examined: APP = 12 mice; NTG = 11 mice. *P <0.05, **P <0.01, and ***P <0.001. n.s. not significant.


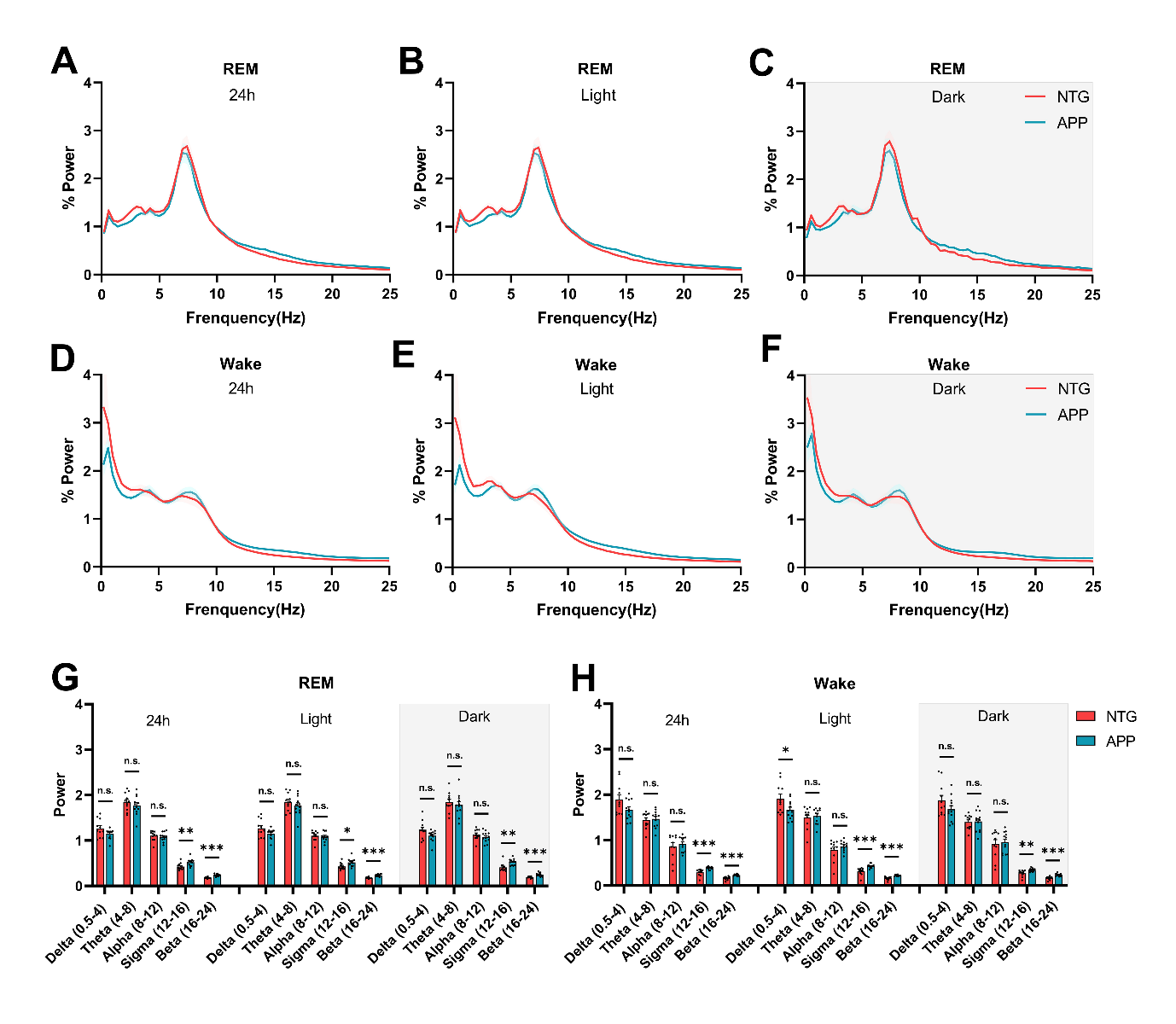


**Supplemental Fig. 2. Optogenetic stimulation of mCherry in absence of ChR2 does not significantly affect the sleep of NTG mice.**

(A) Averaged time spent in each sleep-wake cycle stage (NREM, REM and wake) during 24-hour, 12-hour dark phase and 12-hour light phase of NTG mice before and during stimulation on mCherry.

(B) Time course of the changes in NREM sleep in NTG mice before and during stimulation on mCherry.

(C-E) Relative power spectral density of NREM sleep during 24-hour, 12-hour dark phase and 12-hour light phase of NTG mice before and during stimulation on mCherry.

(F) The average EEG power density in the delta (0.5–4 Hz) and theta (4–8 Hz) bands in NREM sleep during 24-hour, 12-hour dark phase and 12-hour light phase of NTG mice before and during stimulation on mCherry.

(G) The average SWA (0.5–1 Hz) in NREM sleep during 24-hour, 12-hour dark phase and 12-hour light phase of NTG mice before and during stimulation on mCherry.

All data are expressed as means ± standard error. The number of mice examined: n = 7 mice/group.


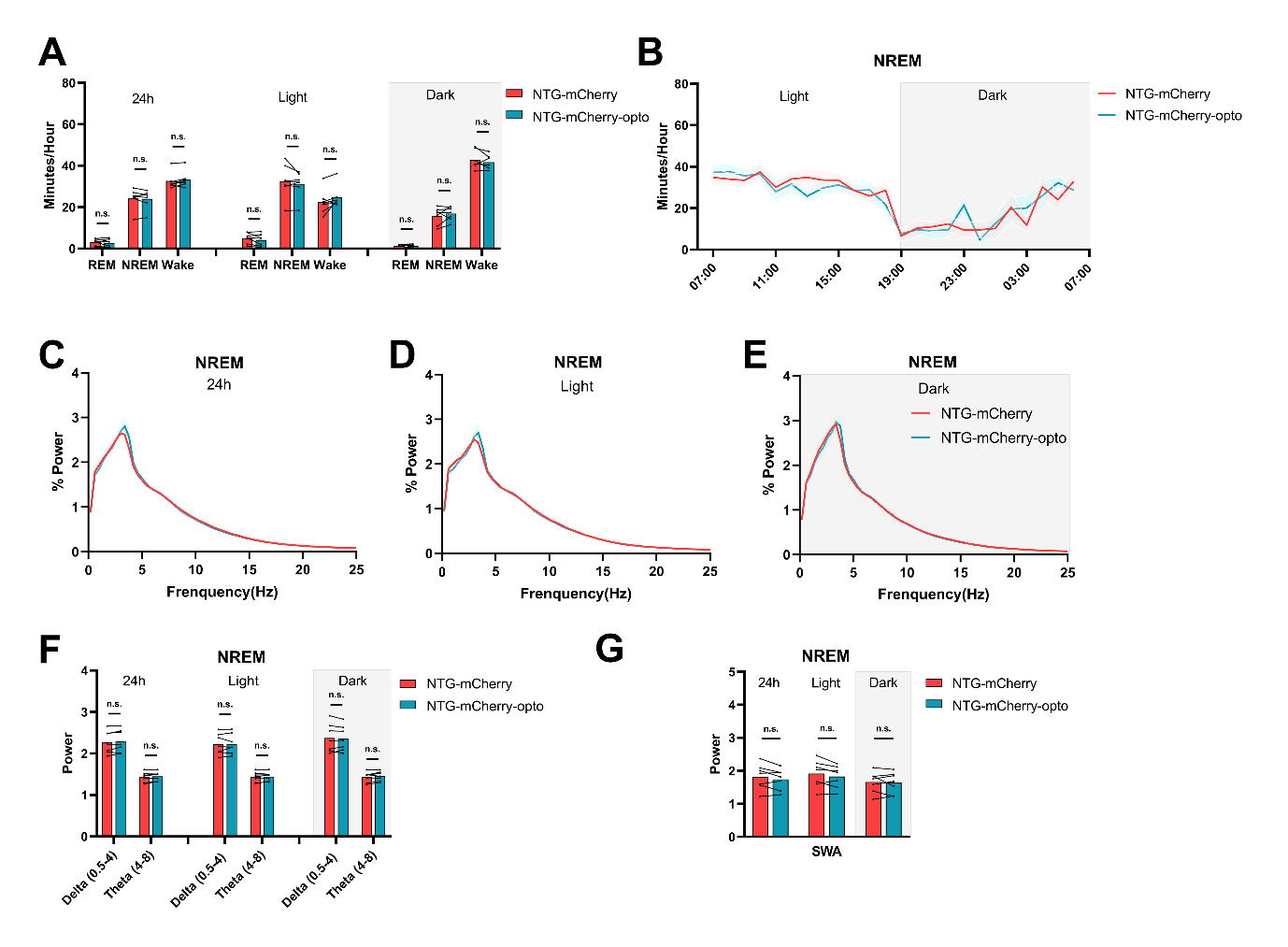


**Supplemental Fig. 3. Optogenetic stimulation of mCherry in absence of ChR2 does not significantly affect the sleep of APP mice.**

(A) Averaged time spent in each sleep-wake cycle stage (NREM, REM and wake) during 24-hour, 12-hour dark phase and 12-hour light phase of APP mice before and during stimulation on mCherry.

(B) Time course of the changes in NREM sleep in APP mice before and during stimulation on mCherry.

(C-E) Relative power spectral density of NREM sleep during 24-hour, 12-hour dark phase and 12-hour light phase of APP mice before and during stimulation on mCherry.

(F) The average EEG power density in the delta (0.5–4 Hz) and theta (4–8 Hz) bands in NREM sleep during 24-hour, 12-hour dark phase and 12-hour light phase of APP mice before and during stimulation on mCherry.

(G) The average SWA (0.5–1Hz) in NREM sleep during 24-hour, 12-hour dark phase and 12-hour light phase of APP mice before and during stimulation on mCherry.

All data are expressed as means ± standard error. The number of mice examined: n = 6 mice/group.


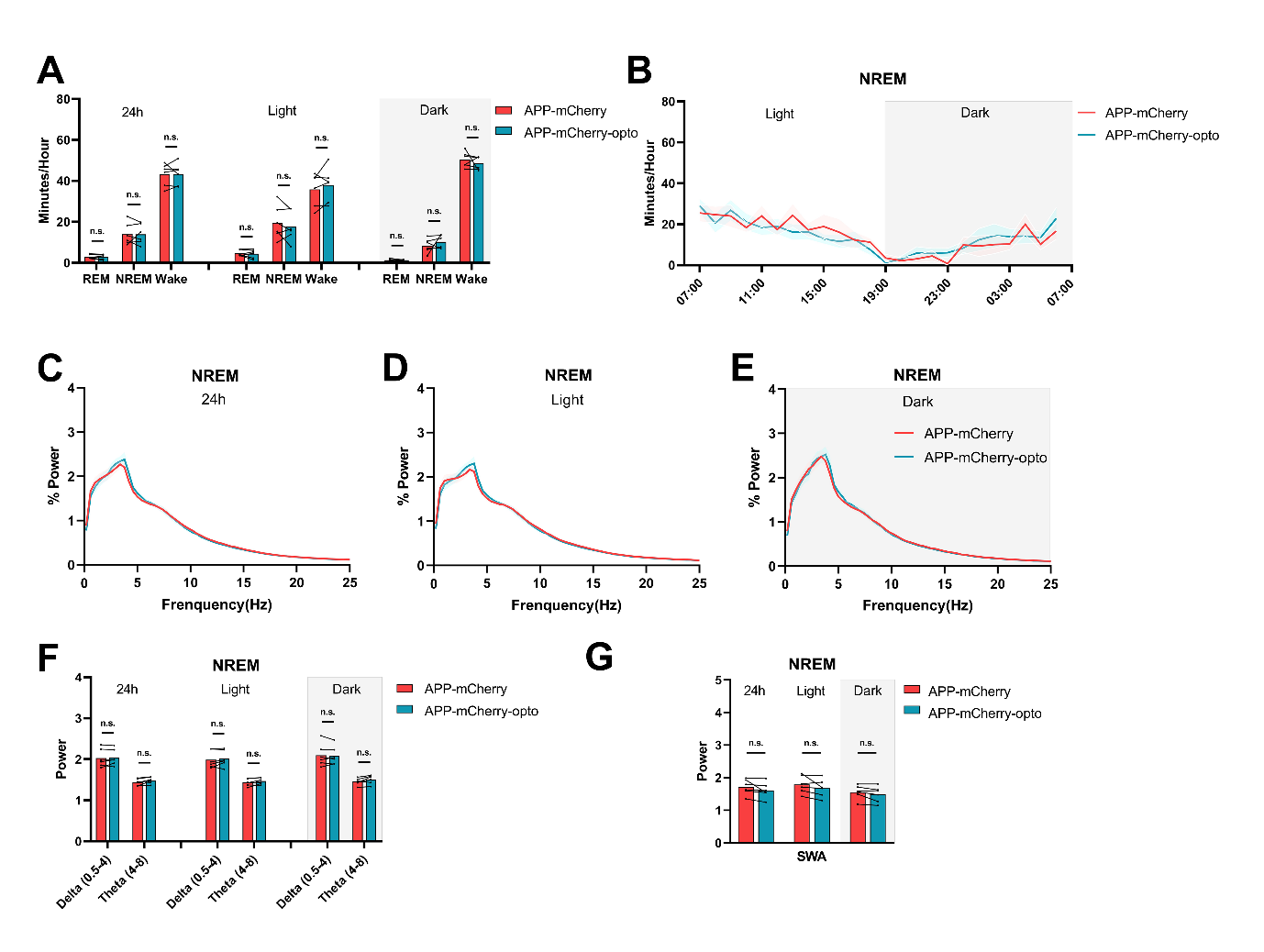


**Supplemental Fig. 4. Sleep measures across groups.**

(A) Averaged time spent in each sleep-wake cycle stage (NREM, REM and wake) during 24-hour in APP mice expression mCherry or ChR2.

(B) The average EEG power density in the delta (0.5–4 Hz) and theta (4–8 Hz) bands in NREM sleep during 24-hour in APP mice expression mCherry or ChR2.

(C) The average SWA (0.5–1Hz) in NREM sleep during 24-hour, 12-hour dark phase and 12-hour light phase in APP mice expressing mCherry or ChR2.

All data are expressed as means ± standard error. The number of mice examined: n = 6 mice/group. *P <0.05. n.s. not significant.


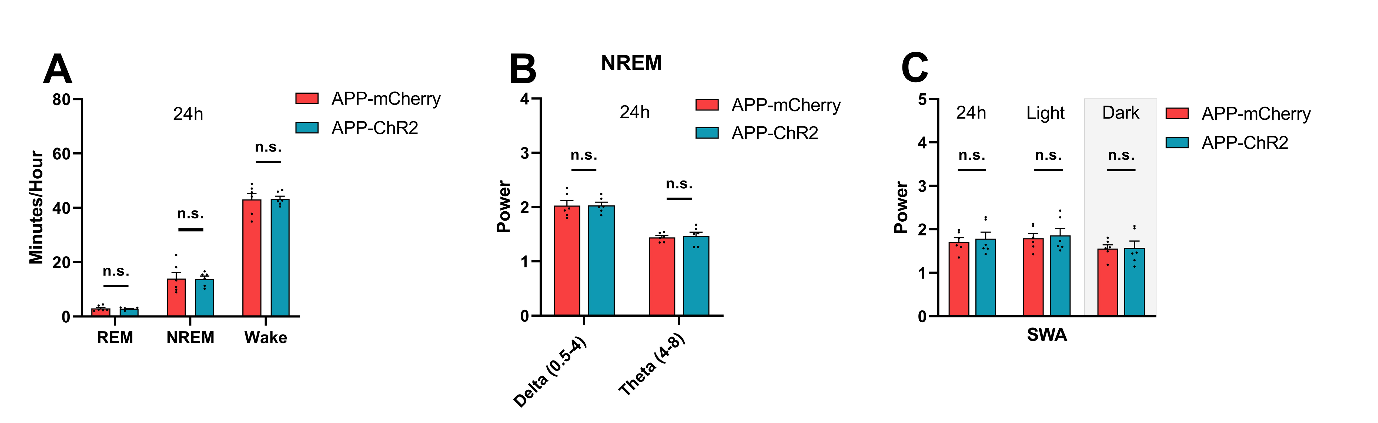


**Supplemental Fig. 5. Expression of ChR2 in absence of optogenetic stimulation does not significantly affect plaque deposition and microglia in APP mice.**

(A-C) 6E10 (A), 82E1 (B) and Methoxy-X04 (C) positive amyloid plaque burden across conditions.

(D) Quantitation of the microglial cell population.

(E) Quantiﬁcation of the Methoxy-X04^+^CD11b^+^CD45^low^ microglia.

All data are expressed as means ± standard error. The number of mice examined: n = 4-6 mice/group. n.s. not significant.


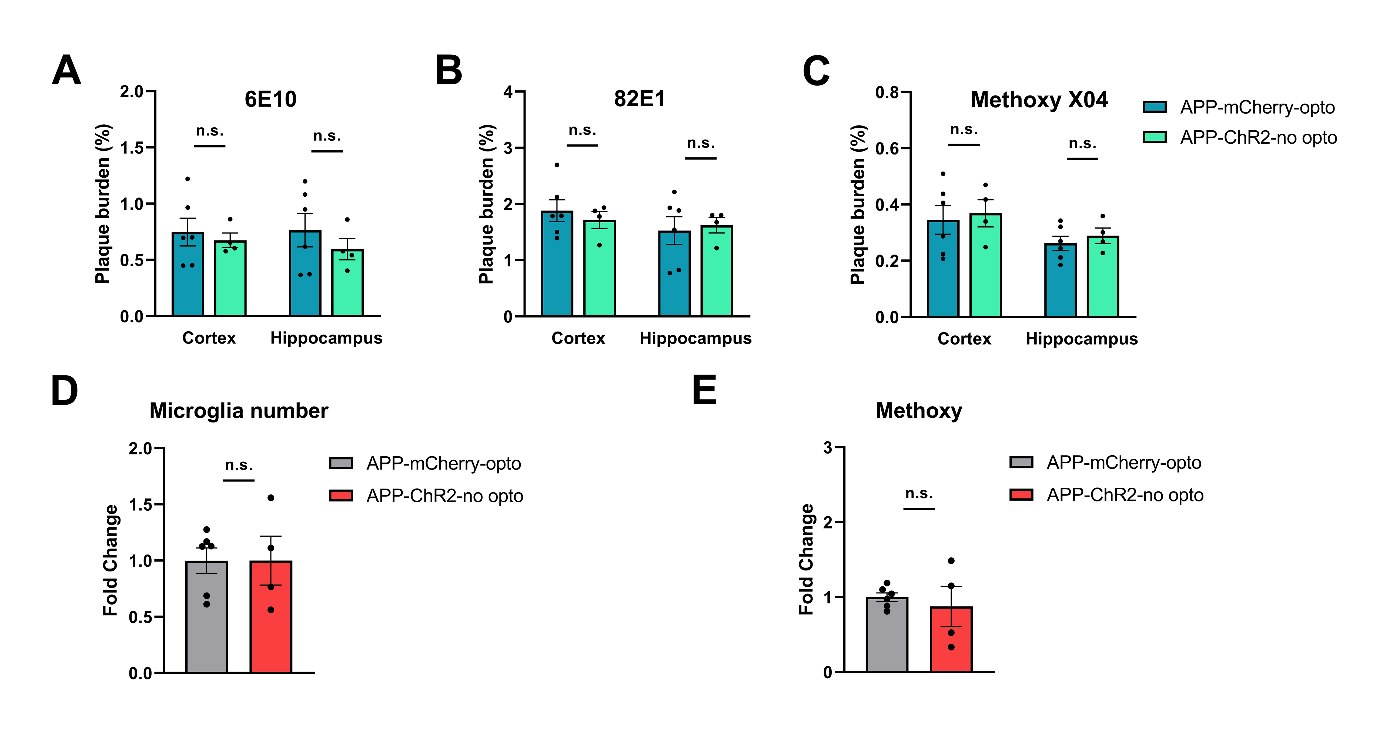


**Supplemental Fig. 6. Effect of chronic optogenetic stimulation of GABAergic neurons on amyloid plaque deposition in amygdala of APP mice.**

(A) Representative images of 6E10, 82E1 and Methoxy-X04 positive amyloid plaques in amygdala within postmortem sections.

(B-D) 6E10 (B), 82E1 (C) and Methoxy-X04 (D) positive amyloid plaque burden across conditions.

All data are expressed as means ± standard error. The number of mice examined: n = 6 mice/group. n.s. not significant. Scale bar: 100 μm.


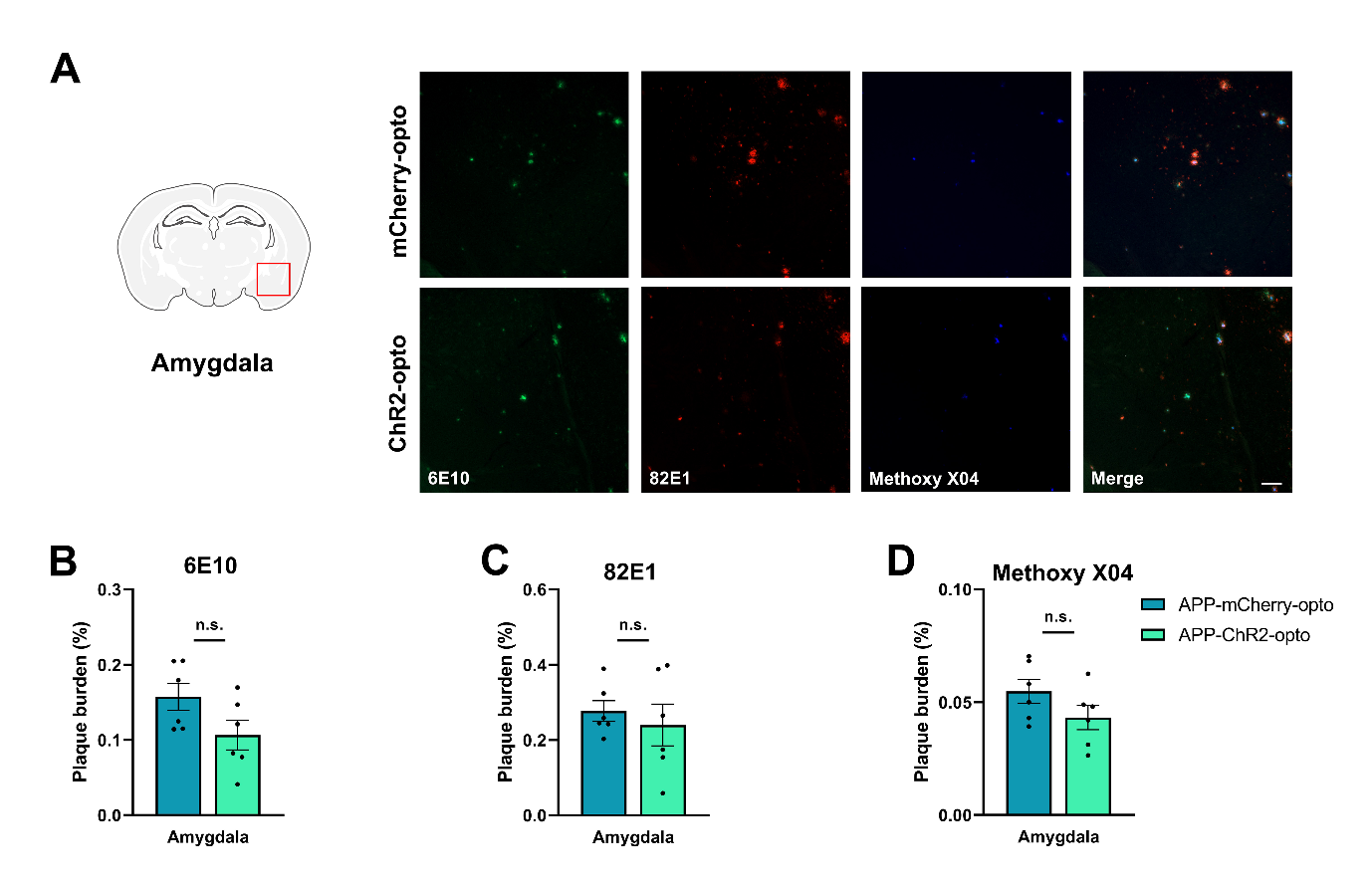


**Supplemental Fig. 7. Chronic optogenetic stimulation of GABAergic neurons in combination with sleep deprivation does not significantly affect sleep, plaque deposition and microglia in APP mice.**

(A) Averaged time spent in each sleep-wake cycle stage (NREM, REM and wake) during 24-hour phase of APP mice before and during optogenetic stimulation and sleep deprivation combination.

(B-D) 6E10 (B), 82E1 (C) and Methoxy-X04 (D) positive amyloid plaque burden across conditions.

(E) Quantitation of the microglial cell population.

(F) Quantiﬁcation of the Methoxy-X04^+^CD11b^+^CD45^low^ microglia.

All data are expressed as means ± standard error. The number of mice examined: n = 3-6 mice/group. n.s. not significant.


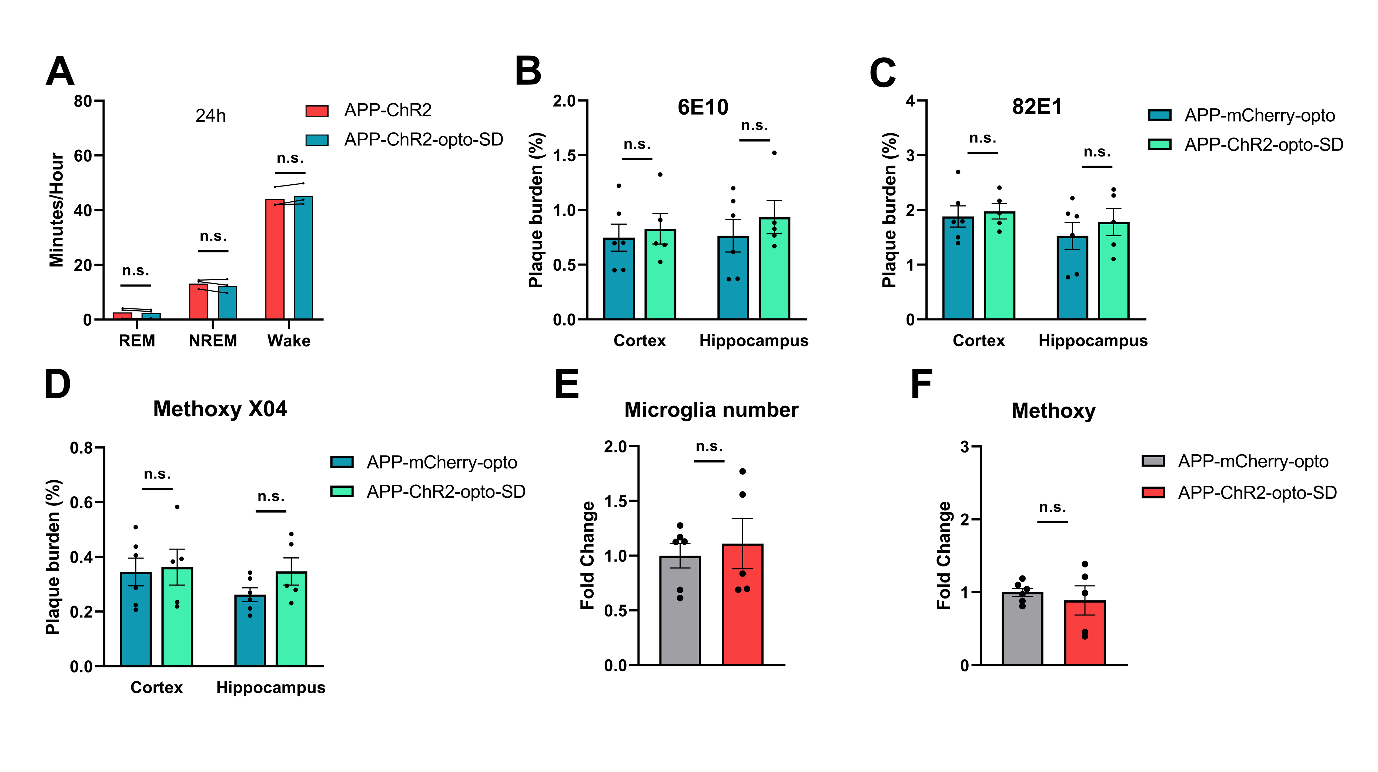


**Supplemental Fig. 8. Chronic optogenetic stimulation of cortical GABAergic neurons at 40 Hz does not significantly affect plaque deposition and microglia in APP mice.**

(A-C) 6E10 (A), 82E1 (B) and Methoxy-X04 (C) positive amyloid plaque burden across conditions.

(D) Quantitation of the microglial cell population.

(E) Quantiﬁcation of the Methoxy-X04+CD11b+CD45low microglia.

All data are expressed as means ± standard error. The number of mice examined: n = 5-6 mice/group. n.s. not significant.


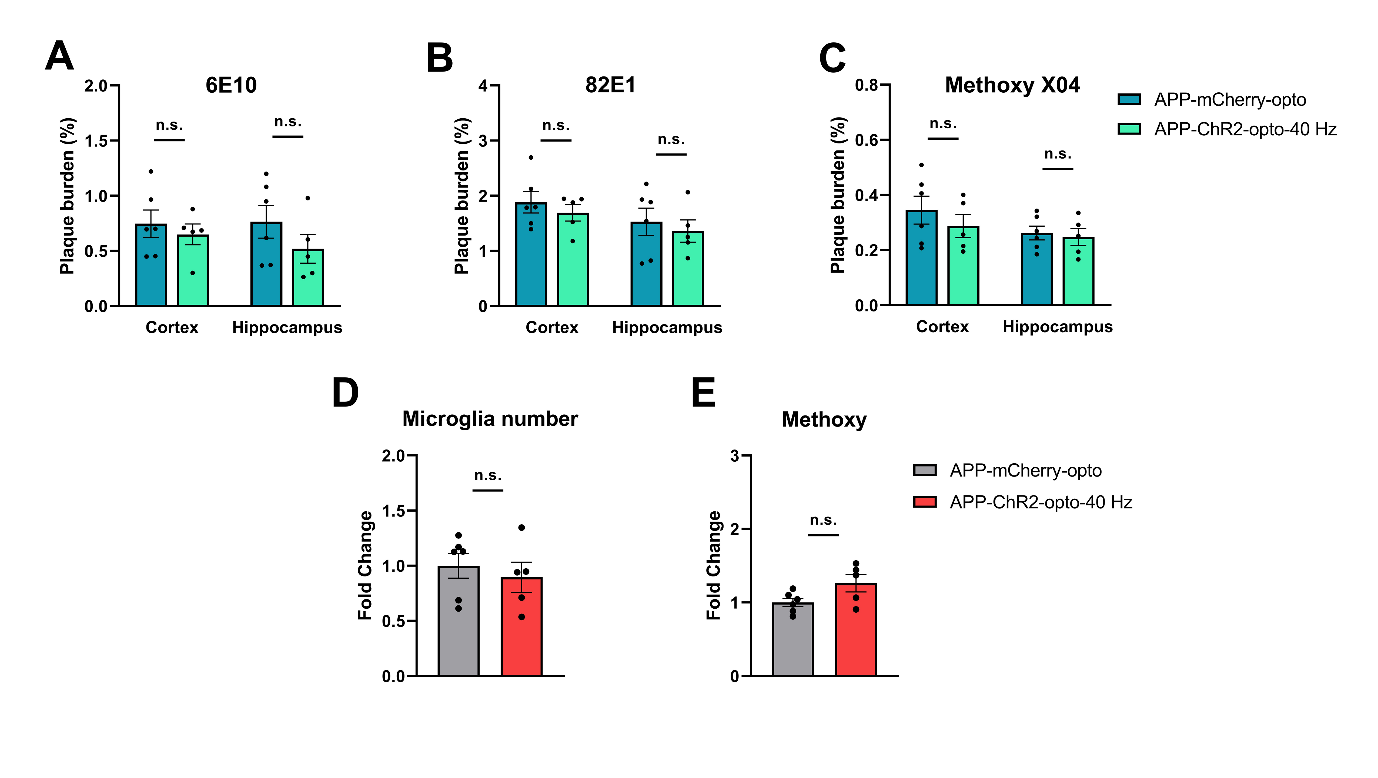

Supplement: Supplementary file 2 — Supplementary Material 2: Supplemental Figures [file 13024_2023_682_MOESM2_ESM.docx]
